# The relationship between physician burnout and depression, anxiety, suicidality and substance abuse: A mixed methods systematic review

**DOI:** 10.3389/fpubh.2023.1133484

**Published:** 2023-03-30

**Authors:** Emer Ryan, Kevin Hore, Jessica Power, Tracy Jackson

**Affiliations:** ^1^Department of Cardiothoracic Anaesthesia, Auckland City Hospital, Auckland, New Zealand; ^2^Usher Institute, University of Edinburgh, Scotland, United Kingdom; ^3^College of Anaesthesiologists of Ireland, Dublin, Ireland; ^4^Department of Anaesthesia, Great Ormonde Street Children's Hospital, London, United Kingdom; ^5^Centre for Global Health, Trinity College, Dublin, Ireland

**Keywords:** burnout—professional, depression, anxiety, suicidal ideation (SI), substance misuse, systematic review

## Abstract

**Introduction:**

The World Health Organization defines burnout as a problem associated with employment, a category distinct from psychological disorders such as depression, anxiety, suicidality and disorders of substance abuse. Evaluating the association between burnout as an occupational exposure and psychological morbidity may indicate that burnout can act as an occupational risk factor for mental ill-health. The systematic review explores this relationship in physicians due to the increased risk in this population and the implications for healthcare delivery.

**Methods:**

A mixed methods systematic review of the literature was conducted across Medline, Cinahl Plus, PsycInfo, Web of Science and The Cochrane Library. Databases were systematically searched using keywords relating to physician burnout and depression, anxiety, suicidality and substance abuse. Identified articles were screened for eligibility by two independent researchers. Data extraction was performed and studies assessed for risk of bias. Quantitative and qualitative results were integrated using a convergent segregated approach and results portrayed as a narrative synthesis.

**Results:**

Sixty-one articles were included in the review. There was notable heterogeneity in the measurement and criteria used to define burnout limiting the assimilation of results. Despite this, all studies that measured the association between depression and burnout reported a significant association. Studies that reported association between burnout and anxiety were similarly uniformly consistent. Most studies that reported the association between burnout and suicidality indicated that a significant association exists however difficulty in measurement of suicidality may have influenced variability of results. The reported association between substance abuse and burnout was more variable, suggesting that any association is likely to be weak or influenced by other variables. Qualitative studies described the manifestations of chronic workplace stress as well as perceived links with psychological morbidity. These included lack of time for work-life balance, the contribution of professional relationships and a culture of invulnerability that exists among physicians.

**Conclusion:**

The systematic review cannot conclude causality but suggests that physician burnout is associated with depression, anxiety and suicidality. Qualitative data provides insight into the nature of this association. The review indicates the need for longitudinal research and provides considerations for intervention strategies to prevent the development and progression of burnout.

**Systematic review registration:**

https://www.crd.york.ac.uk/prospero/display_record.php?ID=CRD42020172938, identifier: CRD42020172938.

## 1. Introduction

Burnout syndrome is a condition caused by excessive workplace stress and is often characterized by the dimensions of emotional exhaustion, depersonalization and reduced personal accomplishment (1, 2). The World Health Organization defines burnout as “a syndrome resulting from chronic workplace stress that has not been successfully managed” (3). As such, physicians and other healthcare workers have been identified as an at-risk group due to the number of occupational factors associated with the profession (4, 5). Studies comparing rates of burnout in physicians to other members of the workforce find consistently higher rates of burnout in physicians (6, 7). Overall prevalence of physician burnout can be difficult to quantify given the heterogeneity definition criteria for burnout within the literature. However, the most commonly quoted prevalence estimates are 50% or greater (8) giving some indication of the scale and gravity of the problem.

Burnout in physicians has well-documented associations with sub-optimal patient care (9) and poor clinical outcomes, as well as absenteeism and decreased productivity (10). Mathematical studies estimating the cost of physician burnout to healthcare systems suggested an overall cost of $4.6 billion within the American healthcare system and $7,600 per physician (11). This estimate does not consider the cost of burnout that is harder to quantify, such as effect on other healthcare staff and disruption to patient continuity of care. The association between burnout and outcomes for the affected individual makes up a smaller proportion of this research subgroup. Many studies that evaluate individuals with burnout, consider burnout as an endpoint as opposed to an exposure or possible risk factor for other outcomes leaving a gap in the literature of the impact of burnout in relation to secondary associations for example of depression, anxiety, substance abuse and suicidality for the physician as an individual (12).

Suicide is among the highest causes of physician mortality and is reportedly the only cause of death where the risk is higher among physicians than the general population (13). Rates of suicide are twice as high among physicians than the general public, with female physicians being between twice and six times as likely to die by suicide than other female groups (14). This contrasts with lower rates of illness such as cardiovascular disease, tobacco-related cancers and stroke (6). It is likely that lower rates of physical illness can be accounted for by knowledge of risk factors, the impact of health-related behavior, symptoms and access to services (7). This poses the question as to why rates of suicide continue to be high despite similar knowledge, with few physicians receiving mental healthcare before their deaths (15). Denial of symptoms, self-diagnosis and treatment, stigma and concerns about career prospects have been hypothesized as possible barriers to help-seeking, although research in this area is limited (16).

Mood disorders such as depression and anxiety which are also highly prevalent among physicians have been identified as important risk factors for physician suicide (16). Lifetime risk of depression among physicians is suggested to be as high as 15% for men and 20–30% for women (17) as compared to reported lifetime risk estimates among the general population of 9% for men and 15% for women (18). Comorbid substance misuse disorders are common among physicians with mood disorders, suicidal ideation or completed suicide (19). Although established, the relationships between substance abuse, mood disorders and suicide are complex, and causation is likely to occur in both directions with some physicians self-medicating due to underlying mood disorders and in others mood disorders may be precipitated by substance misuse (20). Overall rates of alcohol and substance abuse have a similar prevalence to the general population, however severity of addiction at presentation and late presentation are features that are more common in this group. Referral to services is frequently made by concerned colleagues due to absenteeism, intoxication at work or poor work performance (21). Despite late presentation, evidence from intervention programs specifically targeting physicians suggest highly successful treatment rates for those who engage with services (22, 23).

Although a relatively small area of burnout research, occupational stress and burnout have been identified as factors associated with psychiatric morbidity (24, 25). Controversy exists regarding the nature of the relationship between burnout and well-defined illness such as depression, anxiety and substance abuse. For example, researchers have argued that the considerable overlap between features of depression or clinical anxiety and burnout would suggest that they should not be considered as distinct entities (26). Others highlight the importance of the distinction, which avoids pathologizing burnout as it has the potential for modification at an organizational, structural and societal level (27) and should therefore only be considered an occupational risk factor for the development of psychiatric illness. As part of the description in the ICD 11, the WHO categorically states that burnout exists in the context of the workplace and should not be applied to symptoms that occur in other parts of life (3). The objective of this research is to further investigate the relationship between physician burnout and the outcomes of depression, anxiety, substance abuse and suicidality. Clarifying the nature of the association between burnout and depression, anxiety, substance abuse and suicidal ideation may help to identify strategies required to modify this relationship, an effective point of intervention as well as the type of specialist services needed.

There has been an exponential increase in the volume of research conducted into burnout within the last decade (28) and the range of research goals and interests varies considerably. Burnout research can be broadly categorized six subgroups; contributing factors, prevalence, measurement and validation of psychometric tests, interventions and treatment, consequences of burnout and studies aiming to determine the underlying physiological processes or identify biomarkers (28). This research will focus on the consequences of burnout for physicians, exploring the association between burnout and outcomes of depression, anxiety, suicidality and/or substance abuse addressing the gap in the literature for this association within this high-risk group.

### 1.1. Aims and objectives

The aim of this research was to undertake a systematic review of existing literature to answer the research question “*What is the relationship between physician burnout and depression, anxiety, suicidality and substance abuse?*” using a mixed-methods approach to both measure the association between burnout (as an occupational exposure) and each of the specified outcomes and to explore the nature of this association.

The research objectives were:

a) To systemically search the literature to identify articles relating to the association between physician burnout and depression, anxiety, suicidality and/or substance abuse.
b) To critically appraise and assimilate identified studies to describe the association between burnout and outcomes of depression, anxiety, suicidality and/or substance abuse.
c) Explore the nature of any identified associations between burnout and depression, anxiety, suicidality and/or substance abuse through qualitative literature synthesis.

## 2. Methods

The study protocol was designed in accordance with the Preferred Reporting Items of Systematic Review and Meta-analysis-Protocol (PRISMA-P) guidelines (29). The protocol was registered with the International Prospective Register for Systematic Reviews (PROSPERO) (CRD 42020172938). Protocol was adhered to throughout the research process in keeping with PRISMA guidance. Ethical approval was received from Edinburgh University Usher ethics committee.

### 2.1. Research design

A mixed methods systematic review of the literature was carried out. Burnout as a phenomenon occurs as a result of complex interaction between environment, personality and experience (30). To explore the relationship between burnout and depression, anxiety, suicidality or substance misuse it is important not only to measure the degree of association but also explore the perceived links that account for this association through the lens of those affected. A mixed methods approach was chosen so that quantitative findings may investigate and measure the degree of association, while qualitative findings may be used to enrich the understanding of the social processes involved.

### 2.2. Search strategy

The research question was clarified using a PEO format (Population, Exposure, Outcome). The PEO format is considered to be more suitable for research questions relating to etiology and risk than the traditional PICO (population, intervention, comparator, outcome) format (31). A comprehensive selection of search terms for each aspect of the research question were identified during initial scoping review as well as subject heading searches of selected databases. Search terms were adapted for the key concept headings of (i) physicians, (ii) burnout, (iii) depression, (iv) anxiety, (v) suicide, and (vi) substance abuse. Searches were conducted across the following electronic databases; Medline, Cinahl Plus, PsycINFO, Web of Science and the Cochrane Library. Relevant articles were also identified by means of hand-searching reference lists of included studies. The final terms used and search strategy for each database can be seen in Supplementary material 1 limits were set in terms of publication date or type. Limits were set to English Language.

### 2.3. Screening and study selection

Searches of selected databases was performed on February 15th 2020. In keeping with PRISMA guidelines, title/abstract and full text screening were carried out by two independent reviewers, with a third reviewer available for any disagreements should they arise (29). Studies were accepted or rejected based on predefined eligibility criteria as outlined in Table 1, with reasons for exclusion recorded.

**Table 1:** Eligibility criteria.

	**Inclusion criteria**	**Exclusion criteria**
Population	➢ Doctors/physicians ➢ All grades ➢ All specialties ➢ Hospital and non-hospital	➢ Medical students ➢ Healthcare workers other than doctors ➢ Studies that include physicians/doctors but where physicians are not analyzed separately
Exposure	➢ Burnout ➢ Quantitative studies must specify an objective measure of burnout	➢ Occupational stress only without measure of burnout
Outcome	➢ At least one of: (i) Depression (ii) Suicide/suicidal ideation (iii) Anxiety/anxiety disorders (iv) Substance misuse/abuse/addiction. ➢ Quantitative studies must specify an objective measure of outcome	➢ Outcomes other than: (i) Depression (ii) Suicide/suicidal ideation (iii) Anxiety/anxiety disorders (iv) Substance misuse/abuse/addiction
Study design	➢ Primary studies that investigate the relationship between burnout and outcomes: - Quantitative studies must report the *association/correlation* between exposure and outcome - Qualitative studies must explore burnout and at least one outcome	➢ Purely narrative review articles with no original measure of exposure/outcome -Quantitative studies assessing only prevalence of burnout, depression, suicide, anxiety or substance abuse with no measure of correlation/association - Studies assessing interventions to reduce burnout unless baseline measure of association reported
Other	➢ Studies in English or with English translation	➢ Studies in language other than English or with no English translation

### 2.4. Quality assessment

All included studies underwent a quality assessment by two reviewers. Quantitative studies were assessed using the Joanna Briggs Institute (JBI) Critical Appraisal Tool specific to the study design, cross-sectional or cohort study (32). Qualitative studies were assessed using Critical Appraisal Skills Program (CASP) Qualitative Research Assessment Tool (33). Both the JBI and CASP tools allow for objective assessment without scoring systems and studies may be included or excluded based on results. The format is similar in both tools with questions regarding potential areas of bias to be answered as “Yes” “No,” or “Unclear.” For the purposes of this review, studies were deemed low, moderate, or high risk of bias and results were described narratively and in table format.

### 2.5. Data extraction

Data was extracted and recorded using pre-piloted data extraction forms. Using a parallel approach to data extraction, quantitative and qualitative studies were assessed separately using different data extraction tools. For quantitative studies the following data was recorded; (i) bibliographic information, (ii) study design, (iii) study population characteristics—type of physician, stage of training, (iv) number of participants, (v) measure of burnout used, (vi) what outcome(s) were measured, (vii) measure of outcome used and (viii) results (i.e., measure of association). For qualitative studies the following information was recorded; (i) bibliographic information, (ii) setting, (iii) research method, (iv) study aims, (v) number of participants, (vi) themes explored, (vii) data analysis and (viii) authors conclusions.

### 2.6. Evidence synthesis

Evidence was synthesized using a convergent segregated method whereby results were analyzed separately, in parallel and subsequently integrated in a narrative synthesis (34). Aggregation of quantitative results by means of meta-analysis was limited by heterogeneity of studies (35). This was primarily due to variation of population characteristics as well as variability of measurement tools and criteria used to define burnout. A descriptive method was chosen, where results are presented by narrative synthesis. Evidence was then integrated using a configurative analysis whereby themes were compared, linked and juxtaposed between qualitative and quantitative evidence.

## 3. Results

### 3.1. Search results

**Figure 1:**
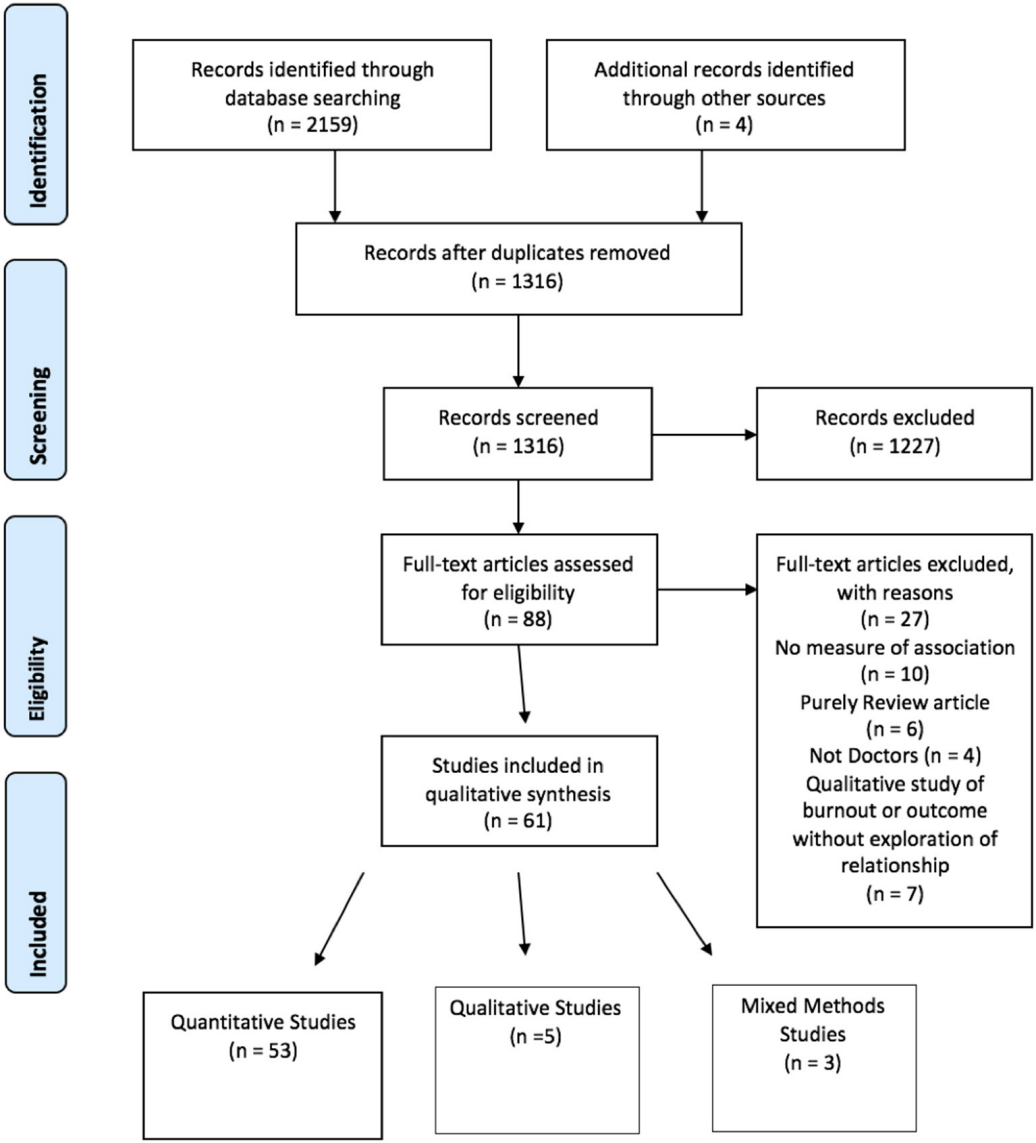
PRISMA - P flow diagram.

The search was carried out on February 15th 2020. Initial searches resulted in 2,159 articles for review. Once duplicates were removed 1,312 articles remained. One thousand three hundred and twelve articles underwent title and abstract screening by two independent reviewers, of which 1,227 were excluded. Three articles were identified from reference list searching and included in full text review. Eighty-eight articles underwent full text screening by both reviewers. Twenty-seven studies were excluded following full text screening for reasons outlined in 1. Sixty-one articles were subsequently included in the systematic review. Of the included studies, 53 used purely quantitative research methods (35–86), five studies contained only qualitative data (87–91) and three studies were designed using mixed methods (92–94). PRISMA P flow diagram of search and included studies can be seen in Figure 1.

### 3.2. Quantitative

#### 3.2.1. Quantitative quality assessment

Quality of included studies containing quantitative data was assessed using Joanna Briggs Institute Critical Appraisal Tools (95). The checklist for cross-sectional studies was used to assess cross-sectional studies and checklist for cohort studies was used to assess the one included cohort study (36). The checklist for cross-sectional studies consists of eight questions, each to be answered yes, no or unclear. In this review studies were given an overall rating based on answers. Studies were considered to be high quality if all questions were answered yes, moderate quality if 6–7 questions were answered yes or any questions answered unclear and low quality if more than two questions were answered no.

Sixteen cross-sectional studies were given an overall rating of high quality, 32 studies were deemed to be of moderate quality and five were given an overall rating of low quality. The one included cohort study was given an overall rating of moderate quality (36). No studies were excluded based on quality, however quality assessment was used to inform the interpretation of results. Quantitative quality analysis results table can be seen in Supplementary Table 2.

##### 3.2.1.1. Study participants

All studies described inclusion criteria that represented the population of interest in the review. Ten studies (36, 37, 43, 46, 57, 60, 63, 68, 77, 78, 84) did not mention survey response rates. No definitive criteria for acceptable survey response rates exist, however 10 included studies (35, 40, 52, 54, 62, 65, 67, 71, 74, 82, 83) had response rates of < 40% which would be uniformly considered low (96). All included studies provided detailed descriptions of participants demographic information.

##### 3.2.1.2. Exposure and outcome measurement

Measurement of exposure was considered low quality in only four studies (43, 73, 75, 82). One study used a single question surrogate adapted from the MBI to assess burnout domains (82). Two studies used measures of overall occupational stress or distress as measures of burnout (73, 75) and one study drew correlations from a larger study and therefore did not describe exposure measurement in detail (43). Studies that used “self-reported” presence or absence of outcome as a means of outcome measurement were assessed as low quality as they lacked external validity.

##### 3.2.1.3. Confounding factors

Thirty-one studies used appropriate methods to account for potential confounding factors (35, 37, 40, 41, 46, 47, 50, 52–54, 57, 58, 60, 62–65, 69, 71–77, 80, 82, 83, 85, 86). Of these one used a control group (44) whereas all others used multivariate analysis to account for confounders.

##### 3.2.1.4. Cohort follow-up

The one included cohort study did not report loss of follow-up over time and only included participants that completed surveys throughout follow-up (36). There was no mention of incomplete data or efforts made to address incomplete response rates, indicating possible selection bias.

#### 3.2.2. Quantitative results

##### 3.2.2.1. Study and population characteristics

Quantitative studies consisted of 54 cross-sectional studies and one cohort study (36). Study populations were well-defined and covered a range of specialties and grades of physicians. Twelve studies did not specify grade or specialty but included all physicians (35, 43, 51–53, 56, 66, 67, 70, 78, 82, 85). One study specifically investigated consultant physicians (63) whereas twenty studies specifically investigated residents, interns or trainees (36, 37, 39–42, 44, 46, 47, 49, 50, 54, 55, 62, 69, 73, 74, 81, 93, 97). Specialties investigated included Internal Medicine (36–39, 57, 69, 75, 77), Psychiatry (38, 41, 61, 79), Orthopedics (40, 71), Obstetrics and Gynecology (42, 58, 74–76, 86), Pediatrics (44, 57, 75, 93), Oncology (45, 64, 92), General Surgery (46, 55, 65, 75, 83), Family Medicine/General Practice (47, 68, 77), Emergency Medicine (48, 57, 73, 81), Intensive care (59, 84), Plastic Surgery (59), Anesthesia (60, 72, 75, 84), Vascular Surgery (59) and Neurology (75). Number of study participants per study ranged from 48 (42) to 7,905 (65). Studies were conducted across 24 countries. The majority were conducted in the USA (36–38, 42, 46–48, 51, 55, 60, 62, 65, 73, 74, 81–83, 86), four studies were conducted in Japan (50, 64, 79, 93), three in each of France (40, 45, 71), Turkey (44, 68, 78) and China (53, 69, 70), two studies in each of Italy (41, 77), Finland (43, 61) and the UK (58, 63) and one study in all remaining countries including the Netherlands (39), Egypt (49), Brazil (52), Hong Kong (35), Lebanon (54), Pakistan (56), Malaysia (57), India (92), Israel (59), Canada (66), Austria (67), Germany (75), Romania (76), Mexico (80), Lithuania (84), and Denmark (85). Participation across all studies was voluntary and subjects were recruited *via* hospital email lists, training college registers, governing bodies and teaching conferences. Among studies that reported response rates, rates ranged from 16% (67) to 100% (69).

##### 3.2.2.2. Measure of exposure

All studies used a validated measurement tool to assess levels of burnout. The Maslach Burnout Inventory (MBI) was the most frequently used burnout assessment tool, used in 46 studies (35–42, 44–53, 55, 57–61, 63–66, 68–72, 74, 76, 78–86, 92, 93). There were notable differences in the interpretation of MBI results with regards classifying and quantifying burnout. The presence of burnout was defined as a dichotomous outcome in 23 studies (35, 36, 38, 39, 46, 48–52, 55, 57–59, 64, 65, 69, 80–84, 86), based on one or all domain cutoff scores. Eleven studies (40, 41, 45, 47, 63, 70–72, 79, 85, 92) categorized levels of burnout as ordinal variables such as low, moderate or high based on overall score (40, 41, 45, 47, 63, 70–72, 79, 85) or burnout domain scores (92). Twelve studies interpreted burnout scores a continuous variable, 10 of which analyzed each domain score separately (37, 42, 44, 53, 60, 61, 66, 68, 74, 76, 78, 93) and two studies (60, 61) used an overall burnout score by combining the results of all domain scores. Other measures of burnout included the Oldenburg burnout inventory used in two studies (62, 77) the Copenhagen Burnout inventory (56), the Burnout Measure (54), the Copenhagen Psychosocial Stress Questionnaire (75), the Health Professional Stress Inventory (73) and the Hamburg Burnout Inventory (67) used in one study each.

##### 3.2.2.3. Depression

Depression as an outcome was investigated by *n* = 42 studies (36–38, 40–42, 44–52, 54–59, 61–64, 66–69, 71–76, 78, 80, 81, 84, 86, 92, 93). Three studies used “self-reported current or history of depression” as a measure of depression (58, 61, 76) whereas all others used validated depression screening or diagnostic questionnaires. The most frequently used questionnaire was the Patient Health Questionnaire (PHQ), used in 14 studies (37, 38, 40, 41, 45, 46, 50, 54, 55, 62, 64, 84, 86, 92). The PHQ is most commonly used in its 9-question form (PHQ9), other forms included the abbreviated PHQ2 and longer PHQ12. The PHQ uses nine questions to address depression severity and is an abbreviated version of the Primary Care Evaluation of Mental Health Disorders Patient Health Questionnaire (PRIME-MD) (98), a diagnostic instrument for common mental disorders. The full PRIME-MD tool was used in five studies (36, 51, 59, 80, 81). Six studies used the Center for Epidemiological Studies Depression Scale (CES-D) (42, 47, 73, 74, 93, 99). Other tools employed included the Beck's Depression Inventory used in four studies (49, 66, 68, 78), the Hospital Depression and Anxiety Scale employed by Karaoglu et al. (44), the Depression, Anxiety and Stress Scale (DASS) used by three researchers (52, 56, 57) and the Harvard National Depression Scale used by Looseley et al. (72).

###### 3.2.2.3.1. Association of burnout and depression

**Figure 2:**
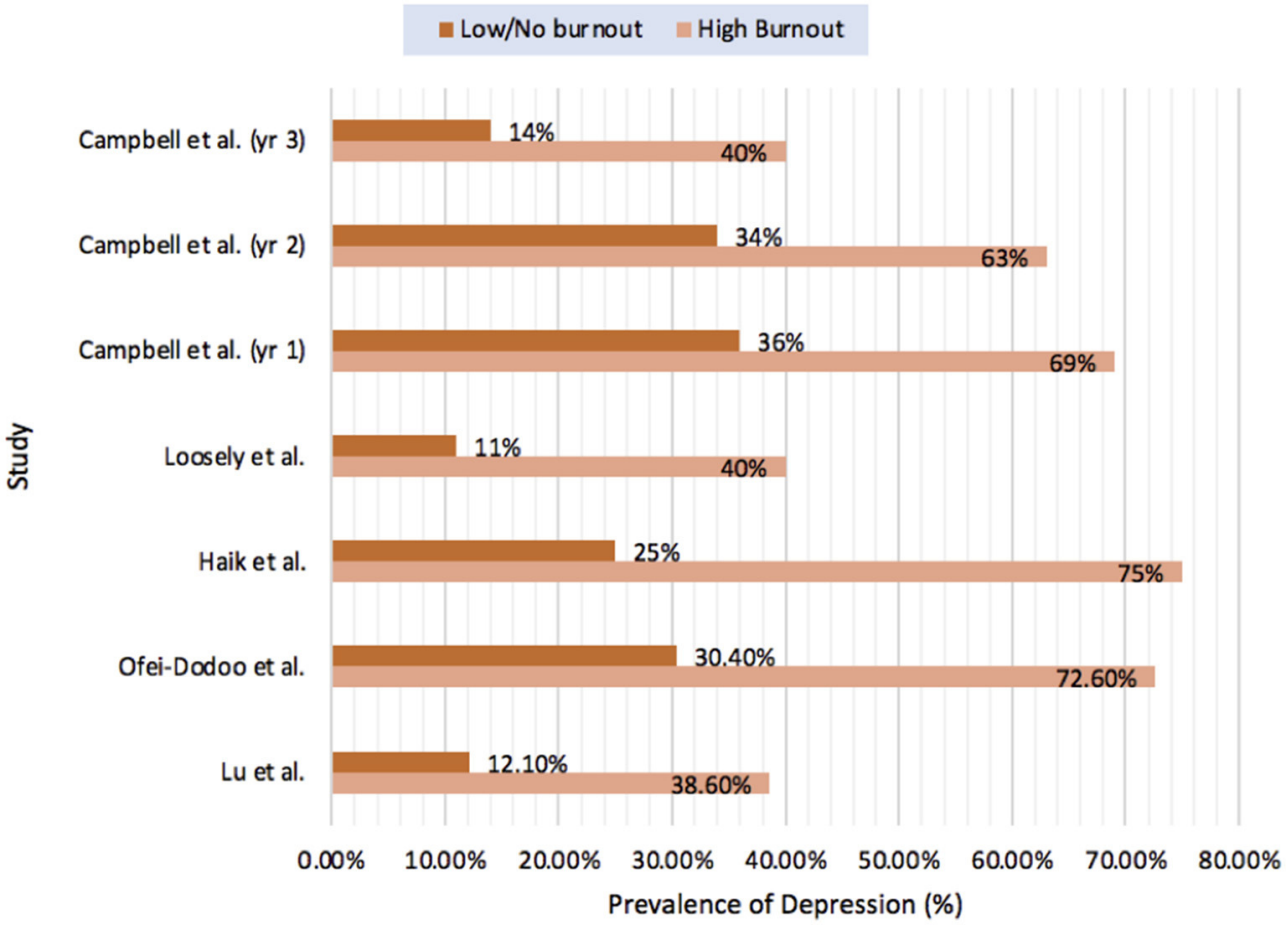
Prevalence of depression in those with burnout compared to those no/low burnout.

Despite notable variation in the measurement and interpretation of burnout score, all 45 studies that investigated the relationship between burnout and depression reported a statistically significant association (36–38, 40–42, 44–52, 54–59, 61–64, 66–69, 71–76, 78, 80, 81, 84, 86, 92, 93). Thirteen studies reported the relationship between presence of burnout and the risk of depression or depressive symptoms as odds ratios (OR) (40, 45, 52, 57, 58, 61, 62, 67, 69, 71, 80, 84, 86) which ranged from 0.89 (57) to 10.68 (69). Studies that measured correlation between overall burnout score or burnout severity with overall depression score reported similar significant results with correlation measuring between *r* = 0.41 (61) and *r* = 0.74 (54, 67). Five studies compared the prevalence of positive depression screens between those with burnout or high burnout and those with low or no burnout and evaluated for statistically significant differences (see Figure 2). The only longitudinal cohort study included in the review (36), aimed to evaluate the association between persistent burnout and depression in internal medicine residents in Colorado during their first 3 years of residency. While they found significantly higher rates of depression in those with persistent burnout they also reported that both burnout and depression scores decreased over time.

Twelve studies evaluated the association between depression and burnout domains separately (44–50, 61, 63, 64, 68, 78, 92). Consistent significant associations were also found between the EE burnout domain and depression whereas other domains were less frequently measured, less consistently significant and where significant showed weaker association. Correlation between burnout domain scores and depression scores was measured by eight studies (44, 47, 49, 50, 63, 64, 68, 78), all of which found statistically significant correlation between EE scores and depression scores which ranged between *r* = 0.16 (64) and *r* = 0.7 (50). Only four studies found significant correlation between DP and depression scores (47, 49, 63, 78), with 3 other studies reporting correlations that were not statistically significant (50, 64, 68). Correlation that was significant was generally lower than that with EE, between *r* = 0.3 (78) and *r* = 0.6 (49). Only 4 studies used PA as a burnout domain during analysis as not all studies considered this to be required for overall measurement of burnout (49, 64, 68, 78). Of these, 3 studies reported significant negative correlation between PA and burnout scores (49, 64, 78) and one reported a correlation that was not significant (68). Similarly, those studies that reported odds ratios between burnout domains and depression reported consistently positive associations between EE and depression (45, 46, 61, 92) with less consistent or weaker association with other domains (45, 46). One of these studies only used the domain EE for multivariate analysis (92) and one study found that only EE and overall MBI score were independently associated with depressive symptoms using logistic regression (61). The studies which measured the association between burnout and depression can be seen in Table 2. Results of the association between burnout and depression categorized by statistical analysis used can be seen in Supplementary Table 3.

**Table 2:** Burnout and depression results.

**References**	**Population**	**Number of participants**	**Measure of association**	**Results**
Ashraf et al. (56)^*^	Physicians (all)	157	Association of work related/client related/person related burnout with depression scores	Person related burnout (χ^2^ = 28.35, *p* < 0.0001) Client related burnout (χ^2^ = 16.08, *p* < 0.05) Work related burnout (χ^2^ = 29.74, *p* < 0.0001)
Becker et al. (74)	Obstetric and gynecology residents	118	Prevalence of low depression scores in low burnout domains	81% with low EE not depressed (*P* = 0.016) 73% with low DP not depressed (*P* = 0.032) 83% with high PA scores not depressed (*P* < 0.0001)
Bernburg et al. (75)	Internal medicine, neurology, surgery, pediatrics, anesthesiology, obstetrics and gynecology	435	Correlation between burnout and depressive symptoms	*R* = 0.25 (*p* < 0.01)
Boo et al. (57)^*^	Internal medicine, pediatric, emergency medicine physicians	313	Odds of depression with high burnout	OR 0.89 (*p* = 0.007)
Bourne et al. (58)^*^	Obs/Gyn residents and consultants	3,102	Odds of depression with burnout	OR 4.05 (95%CI 3.26–5.04)
Campbell et al. (36)	Internal medicine residents	86	Compared prevalence of Depression in those with vs. without Burnout (over 3 years)	Year 1: 69 vs. 36% (*p* < 0.002) Year 2: 63 vs. 34% (*p* < 0.007) Year 3: 40 vs. 14% (*p* < 0.005).
Carter et al. (37)	Medical residents	107	Correlation between burnout domain scores and depression scores	EE: *r* = 0.63 DP: *r* = 0.5 PA *r* = −0.43
Chaukos et al. (38)	Medical and psychiatry residents	68	Compared depression scores in those with burnout vs. no burnout	4.9 ± 5.8 vs. 1.8 ± 2.5 (*p* = 0.035)
Daruvala et al. (92)^*^	Oncology physicians—surgical/medical/radiation oncology	114	Odds of depression with low or high EE Odds of depression or anxiety with low or high EE	OR 2.7 (*p* = 0.09) OR 4.2 (*p* < 0.00001)
Faivre et al. (40)^*^	Orthopedic residents	107	Odds of depression in those with moderate/severe burnout	OR 19.3 (p = 0.0048)
Faivre et al. (71)^*^	Trauma and orthopedic surgeons	441	Odds of depressive symptoms with burnout	OR = 6.3 (*p* = 0.0006)
Ferrari et al. (41)^*^	Psychiatric residents	108	Correlation between depression scores and domain scores.	EE: *r* = 8.4, (*p* = 0.00) Cynicism: *r* = −4.00 (*p* = 0.00).
Govardhan et al. (42)^*^	Obstetrics/gynecology residents	49	Correlation between high depression scores and domain scores	Correlation with high DP: *P* = 0.019 Correlation with high EE: *P* < 0.001
Haik et al. (59)	Burn physicians, plastic surgeons, intensive care physicians	55	Comparison of prevalence of depression with burnout vs. without burnout	75 vs. 25% (*p* < 0.00001)
Iorga et al. (76)	Obstetrics/gynecology physicians	116	Compared mean numbers of those with depression in those with and without positive burnout domain scores	EE: 31 vs. 21 (*p* = 0.002) DP: 9.93 vs. 6.79 (*p* = 0.033) PA: 31.86 vs. 37.47 (*p* = 0.015)
Janko et al. (62)^*^	Vascular surgery trainees	177	Odds of moderate/severe depression with high burnout	OR 2 (*P* < 0.01)
Karaoglu et al. (44)^*^	Pediatric residents (and control group)	74	Correlation between depression and burnout domain scores	EE and depression *r* = 0.65
Khan et al. (63)^*^	Consultants (all)	593	Correlation between depressive symptoms and EE and DP	EE: *r* = 0.61 (*p* < 0.01) DP: *r* = 0.40 *(*p < 0.01)
Korkeila et al. (61)	Psychiatrists/child psychiatrists	3,313	Correlation between depression and overall burnout score	*R* = 0.41 (*p* < 0.001)
Lazarescu et al. (45)^*^	Radiation oncologists	242	Odds of depression with moderate/severe burnout or positive domain scores.	Moderate/severe burnout—OR: 2.96 (*P* < 0.001). EE—OR: 4.7 (*P* < 0.001) PA—OR: 2.2 (*P* = 0.003)
Lebares et al. (46)^*^	General surgical residents	566	Odds of severe depression with high EE or DP scores	EE: OR 4.8163 (*p* < 0.0001) DP: OR 2.3557 (*p* < 0.0009)
Lebensohn et al. (47)^*^	First year family medicine residents	168	Correlation between depression scores and EE and DP scores	EE *r* = 0.584 (*p* < 0.001) DP *r* = 0.518 (*p* < 0.001)
Looseley et al. (72)^*^	Anesthesiologists	397	Comparison of prevalence of depression risk in high burnout risk vs. low burnout risk groups	40 vs. 11% (*p* < 0.0001)
Lu et al. (48)	Year 2–4 and attending emergency physicians	77	Prevalence of burnout in those with positive depression screen compared to those without	38.6 vs. 12.1% (*p* = 0.011)
Mampuya et al. (64)	Radiation oncologists	87	Correlation between burnout domains and psychological morbidity (depression)	EE: *r* = 0.16 (*p* < 0.01) PA: *r* = −0.1 (*p* < 0.01)
Mikalauskas et al. (84)^*^	Anesthetists and intensive care physicians	220	Odds of burnout in those with depression	OR 10.3 (*p* < 0.01)
Mohammed et al. (49)	Resident physicians	84	Correlation between severity of depression and domain scores	EE: *r* = 0.61 (*p* < 0.001). DP: *r* = 0.63 (*p* < 0.001). PA: *r* = −0.56 (*p* < 0.001)
Nishimura et al. (50)	Post-graduate year 1 and 2 resident physicians	39	Correlation between overall depression score and EE and DP scores (across 3 years)	EE: *r* = 0.615 (*p* < 0.001) T1 *r* = 0.706 (*p* < 0.001) T2 *r* = 0.601 (*p* < 0.01) T3 DP: *r* = 0.279 (*p* < 0.086) T1; *r* = 0.047 (*p* < 0.817) T2 *r* = 0.176 (*p* < 0.445) T3
Nomura et al. (93)	Pediatric residents	41	Comparison of mean EE and DP scores in those with high depression scores vs. those without	EE: 15.6 vs. 12.5 (*p* < 0.02) DP: 13 vs. 9.8 (*p* < 0.01)
Ofei-dodoo et al. (51)^*^	Physicians (all)	197	Compared prevalence of depression in those with burnout vs. those without	72.6 vs. 30.4% (*p* < 0.001)
Pasqualucci et al. (52)^*^	Physicians (all)	606	Odds of depression with burnout	OR = 2.7, CI = 1.7–4.1 (*p* < 0.000)
Rath et al. (86)^*^	Obstetrics/gynecology physicians	369	Odds of positive screen for depression in those with burnout	OR 7.34 (*p* < 0.0001)
Sahin et al. (78)	Physicians (all)	158	Correlation between depression scores and burnout domain scores	EE: *r* = 0.516 (*p* < 0.0001) DP: *r* = 0.311 (*p* < 0.0001) PA: *r* = −0.218 (*p* < 0.0001)
Talih et al. (54)^*^	Interns and residents (all)	118	Correlation between severity of burnout scores and depression scores	*r* = 0.72 (*p* < 0.001)
Thommassen et al. (60)	Physicians (all)	131	Correlation between depression and burnout domain scores	EE: statistically significant correlation (*P* < 0.0001), DP: a weaker correlation (*P* < 0.08) PA: no correlation
Toral-villanueva et al. (80)^*^	Junior doctors	312	Odds of depression in those with burnout	OR 5.6 (95% CI 3.3–9.5)
Whitely et al. (73)	Emergency medicine residents	486	Correlation between burnout scores and depression scores	*R* = 0.67 (*P* < 0.0001)
Williamson et al. (81)	Emergency medicine residents	334	Compared mean burnout domain scores of those that screened positive for depression vs. those that did not	EE: 26.8 vs. 17.8 DP: 15.3 vs. 11.2 PA: 36.4 vs. 40.8
Williford et al. (55)^*^	Surgical residents	92	Comparison of Depression score severity in those with burnout vs. those without	Increase of 6 points on depression score with burnout: coefficient [SE = 6.08 (1.41)] (*P* < 0.001)
Wurm et al. (67)	Physicians (all)	5,897	Odds of major depression with mild/moderate/severe burnout. Correlation between burnout score and depression score	Mild: OR 2.99 (95% CI 2.21–4.06) Moderate: OR 10.14 (95% CI 7.58–13.59) Severe: OR 46.84 (95% CI 35.25–62.24) *R* = 0.74 (*p* < 0.001)
Yilmaz et al. (68)^*^	Family physicians	343	Correlation between depression and domain scores	EE: *r* = 0.41 (*P* = 0.0001) Not significant association with depersonalization and PA
Zhang et al. (69)	Internal medicine residents	159	Odds of depression with burnout (domains also analyzed separately)	OR 10.68 (*p* < 0.00001) Significantly associated with EE and DP but not PA

^*^Assessed multiple outcomes.

##### 3.2.2.4. Anxiety

Anxiety as an outcome was measured by *n* = 12 studies (44, 46, 52–54, 56–58, 63, 68, 70, 92). Only two studies assessed anxiety as their primary outcome (53, 70) both of which used the Zung Self-rated Anxiety Scale as a measure of anxiety symptoms. One study used ‘self-reported symptoms' as a measure of outcome with all others using validated anxiety questionnaires. The Depression, Anxiety and Stress Scale (DASS) was used in two studies (56, 57). Other tools used to measure anxiety included the Hospital Depression and Anxiety Scale used by Karaoglu et al. (44), the Speilberg State Trait Anxiety Index and State trait personality Index used by Lebares et al. (46) and Khan et al. (63), respectively, and the Beck's Anxiety Inventory used in one study (68).

###### 3.2.2.4.1. Association of anxiety and burnout

Twelve studies assessed the relationship between burnout or burnout domains and anxiety (44, 46, 52–54, 56–58, 63, 68, 70, 92), six of which assessed burnout domains separately (44, 46, 63, 68, 92, 100). Although a fewer number of studies, similar to results for depression, all studies found significant association between burnout and anxiety or the EE domain and anxiety. Other burnout domains were less consistently significant.

Six studies investigated the association between presence of burnout or overall burnout score and anxiety symptoms, all of which reported a significant association. Three of these studies reported the risk of anxiety in the presence of burnout as odds ratios which ranged between 1.08 (57) and 3.95 (58). Two studies measured the correlation between burnout scores and anxiety scores, both of which reported similar correlation of *r* = 0.46 (54) and 0.47 (70).

Six studies used separate burnout domains and evaluated the association of at least one domain with anxiety disorders or symptoms. The largest included study aimed to investigate anxiety disorders and related factors among 1,134 Chinese physicians (53) and found significant correlation between both EE and DP and anxiety disorders. The studies which measured the association between burnout and anxiety can be seen in Table 3. Results of association categorized by statistical analysis used can be seen in Supplementary Table 4.

**Table 3:** Burnout and anxiety results.

**References**	**Population**	**Number of participants**	**Measure of association**	**Results**
Ashraf et al. (56)^*^	Physicians (all)	157	Association of related/client relate/person related burnout with anxiety scores	Person related burnout (χ^2^ = 30.63, *p* < 0.0001).
Boo et al. (57)	Internal medicine, pediatric, emergency medicine physicians	313	Odds of anxiety with high burnout	OR 1.079 (*p* = 0.08)
Bourne et al. (58)^*^	Obs/Gyn residents and consultants	3,102	Odds of anxiety with burnout	OR 3.59 (95%CI 3.07–4.21)
Daruvala et al. (92)	Oncology physicians—surgical/medical/radiation oncology	114	Odds of anxiety with low or high EE Odds of depression or anxiety with low or high EE	OR 1.67 (*p* = 0.3) OR 4.2 (*p* < 0.00001)
Karaoglu et al. (44)	Pediatric residents (and control group)	74	Correlation between anxiety and burnout domain scores	EE and anxiety *r* = 0.74
Khan et al. (63)	Consultants (all)	593	Correlation between anxiety symptoms and EE and DP	EE: *r* = 0.57 (*p* < 0.01) DP: *r* = 0.40 (*p* < 0.01)
Lebares et al. (46)^*^	General surgical residents	566	Odds of high anxiety with high EE or DP scores	EE: OR 7.2490 (*p* < 0.0001) DP: OR 2.9767 (*p* < 0.0001)
Pasqualucci et al. (52)	Physicians (all)	606	Odds of anxiety with burnout	OR = 2.5, CI = 1.7–3.7 (*p* < 0.000)
Sun et al. (53)	Physicians (all)	1,134	Correlations between anxiety disorder and EE and Cynicism	EE: *r* = 0.46 (m + f) (*p* < 0.01), Cynicism: *r* = 0.49 (m) *r* = 0.51 (f) (*p* < 0.01)
Talih et al. (54)^*^	Interns and residents (all)	118	Correlation between severity of burnout scores and anxiety scores	*r* = 0.47 (*p* < 0.001).
Yilmaz et al. (68)	Family physicians	343	Correlation between anxiety and domain scores	EE: *r* = 0.34 (*P* = 0.001) Not significant association with depersonalization and PA
Zhou et al. (70)	Physicians (all)	1,129	Correlation between anxiety and burnout symptoms	*R* = 0.45 (*p* < 0.001)

^*^Assessed multiple outcomes.

##### 3.2.2.5. Suicidal ideation

Suicidal ideation or suicidal risk and its association with burnout was assessed by *n* = 16 studies (35, 39, 41, 45, 46, 51, 54, 55, 58, 61, 65, 71, 72, 77, 79, 86). The presence or absence of suicidal ideation as a self-reported binary outcome was used by five studies (35, 39, 58, 61, 79). Pompili et al. (77) used the Beck's Hopelessness Index as a measure of suicide risk, a 20-question scale that measures feeling of hopelessness which has been shown to correlate with suicidal risk (101). Other tools used included the suicidal ideation component of the PHQ used in six studies (45, 46, 51, 54, 55, 71), a Suicide Ideation and Behavior Questionnaire (79) and a suicidal ideation question from the Meehan Inventory (65) were used in one study each.

###### 3.2.2.5.1. Association of suicidal ideation and burnout

Fifteen studies investigated the relationship between suicidality and burnout (35, 39, 41, 45, 46, 51, 54, 55, 58, 65, 71, 72, 77, 79, 86). Of these only Siu et al. (35) reported on both suicidal ideation and previous suicide attempts. Ten studies reported significant association (35, 39, 45, 46, 51, 54, 58, 65, 77, 86). Four studies (40, 41, 61, 72) reported higher levels of suicidal ideation in those with burnout but without a measure of association or correlation. One study found no statistically significant association between burnout and suicidal ideation (55).

Only three studies were designed with the primary aim of investigating suicidal ideation or risk (39, 65, 77) and each of these reported a significant positive association with burnout or a burnout domain. The largest study investigated 7,900 surgeons and reported that for each point increase in EE and DP scores and each point decrease in PA score participants were 5.7 to 10% more likely to report suicidal ideation (65). They also reported that the increase in prevalence of suicidal ideation increased in relation to severity of burnout and that this relationship was independent of depressive symptoms. Van der Heijdan et al. (39) reported increased suicidal ideation among 2,000 Dutch medical residents with burnout (20.7 vs. 7.6% *p* < 0.0001) as well as significant correlation between burnout domains and suicidal ideation (*r* = 0.25, *p* < 0.001; *r* = 0.17, *p* < 0.001; *r* = −0.07, *p* < 0.01 for EE, DP, and PA, respectively). Siu et al. (35) reported a statistically significant higher percentage with suicidal ideation in those with “high” burnout levels compared to “low” burnout levels (10% vs. 2.7, *p* = 0.03) however there had were no suicidal acts reported by any participant. The studies which measured the association between burnout and suicidality can be seen in Table 4.

**Table 4:** Burnout and suicidality results.

**References**	**Population**	**Number of participants**	**Measure of association**	**Results**
Bourne et al. (58)^*^	Obs/Gyn residents and consultants	3,102	Odds of suicidal thoughts with burnout	OR 6.37 (95% CI 3.95–10.7)
Faivre et al. (71)	Trauma and orthopedic surgeons	441	Prevalence of suicidal ideation with burnout	8.6% of those with burnout reported suicidal ideation
Ferrari et al. (41)	Psychiatric residents	108	Rate of suicidal ideation measured in those with burnout	High rate of suicidal ideation but no measure of correlation.
Lazarescu et al. (45)	Radiation oncologists	242	Odds of Suicidal Ideation with moderate/severe burnout or positive domain scores.	Moderate/Severe burnout- OR: 0.46 (*P* = 0.01). EE: OR: 2.9 (*P* = 0.002).
Lebares et al. (46)	General surgical residents	566	Odds of suicidal ideation with high EE or DP scores	EE: OR 5.7840 (*p* < 0.0001) DP: OR 2.1827 (*p* < 0.0165)
Looseley et al. (72)^*^	Anesthesiologists	397	Prevalence of suicidal ideation in burnout groups	2.6% reported suicidal ideation
Ofei-dodoo et al. (51)	Physicians (all)	197	Compared prevalence of suicidal ideation in those with burnout vs. those without	100 vs. 46.9% (*p* < 0.01)
Pompili et al. (77)	Internal medicine physicians and GPs	134	Correlation between burnout domain scores and hopelessness scores (marker for suicide risk)	Exhaustion: *r* = 0.2 (*p* < 0.05) Disengagement: *r* = 0.22 (*p* < 0.05)
Rath et al. (86)^*^	Obstetric/gynecology physicians	369	Odds of screening positive for suicidal ideation in those with burnout	OR 4.92 (*p* < 0.001)
Shanafelt et al. (65)	Surgeons	7,905	Correlation between burnout domains and suicidal ideation	EE: OR 1.069 (*P* < 0.001) DP: OR 1.109 (*P* < 0.001) PA: OR 1.057 (*P* < 0.001)
Siu et al. (35)^*^	Physicians (all)	226	Presence of suicidal ideation in those with high burnout vs. those with low burnout	10 vs. 2.6 % (*p* = 0.03).
Talih et al. (54)	Interns and residents (all)	118	Likelihood of suicidal ideation in those with burnout	Burnt-out residents more likely to have suicidal ideation: χ^2^ = 9.4 (*p* = 0.002)
Tateno et al. (79)	Psychiatric trainees	95	Compared differences in presence of suicidal ideation in those with and without positive burnout domains	No significant differences in presence of suicidal ideation on all domains
Van der Heijden et al. (39)	Medical residents	2,115	Presence of Suicidality in those with moderate Burnout vs. those with no burnout	20.5 vs. 7.6% (*p* < 0.001)
Williford et al. (55)^*^	Surgical residents	92	Comparison of presence of suicidal ideation in those with burnout vs. those without	No significant association between burnout and suicidal ideation *P* = 0.11

^*^Assessed multiple outcomes.

##### 3.2.2.6. Alcohol and substance misuse

The association between alcohol or substance misuse and burnout and analyzed by *n* = 16 studies (35, 42, 43, 46, 47, 54, 58, 60, 62, 72, 80, 82–86). The most frequently used measurement tool was the Alcohol Use Disorders Identification Test (AUDIT) which was employed by six studies (46, 54, 80, 82, 83, 85). AUDIT contains 10 questions that relate to alcohol consumption and alcohol consumption behavior and acts as a tool for identifying harmful alcohol consumption (102). Other outcome measures included the CAGE questionnaire, a four-question screening tool used to identify harmful drinking habits (103), which was used in two studies (84, 86). One study used the Alcohol Behavior Index (43) and one used validated single question screening tool (62). Self-reported substance use or alcohol consumption questionnaires with cutoff scores given for harmful consumption was used in two studies. Substance Misuse was assessed by four studies (54, 58, 80, 82), two of which used the Alcohol, Smoking and Substance Involvement Screening Tool (ASSIST) while the other two studies used self-reported use of substances.

###### 3.2.2.6.1. Alcohol and substance misuse

Of the 16 studies that explored the relationship between burnout and substance misuse, 14 studies reported the association between alcohol consumption (35, 42, 43, 46, 47, 54, 62, 72, 80, 82–86) and burnout while only four studies measured the association with other substances (58, 60, 80, 82). One study specifically mentions cannabis use (60) otherwise specific substances were not named. Only one study reported a statistically significant association between abuse of substances other than alcohol and burnout (58).

Fourteen studies reported the relationship between alcohol misuse and burnout and eight of these reported no significant association. Inconsistent results suggest that any association that exists is likely to be weak or subject to other confounding variables. The three largest studies, that were designed specifically to investigate the association between burnout and alcohol misuse did however report a significant positive association (43, 82, 83). The largest included study was a cross-sectional analysis of over 7,000 surgeons which found that significantly more participants with burnout also had symptoms of alcohol misuse (29.6 vs. 25% *p* < 0.0001) and alcohol dependence (34.9 vs. 25% *p* < 0.00001) than those without (82). This study also reported a significant increase in the alcohol misuse or dependence with increasing frequency of features of EE and DP. Similarly, a large cross-sectional analysis designed to investigate the association between alcohol misuse and burnout domains reported positive association between burnout and risky alcohol behavior (OR 1.89 *p* < 0.014) and significant associations with each burnout domain, the largest of which being with DP (OR 2.23 *p* < 0.00001) (85). This study also investigated the association between burnout and alexithymia, or the inability to identify and describe feelings (104) and found a significant association. The findings suggested that alexithymia acted as a mediator between burnout and alcohol misuse, particularly between DP and alcohol misuse. The studies which measured the association between burnout and misuse can be seen in Table 5.

**Table 5:** Burnout and substance abuse results.

**References**	**Population**	**Number of participants**	**Measure of association**	**Results**
Bourne et al. (58)^*^	Obs/Gyn residents and consultants	3,102	Odds of substance abuse with burnout	OR 2.57 (95%CI 1.71–3.89)
Govardhan et al. (42)	Obstetric/gynecology residents	49	Association between alcohol misuse and burnout	No association with alcohol.
Hyman et al. (60)	Anesthesiologists	170	Prevalence of Substance and Alcohol Abuse in those with burnout vs. those without	No increase in substance or alcohol abuse
Janko et al. (62)	Vascular surgery trainees	177	Odds of alcohol abuse with high burnout	No significant association with alcohol abuse
Juntunen et al. (43)	Physicians (all)	2,671	Correlation between high domain scores and Alcohol misuse	Positive correlation with EE and DA, negative correlation with PA
Lebares et al. (46)^*^	General surgical residents	566	Risk of alcohol misuse with high EE or DP scores	Alcohol misuse was not associated with high EE or DP scores
Lebensohn et al. (47)	First year family medicine residents	168	Association between alcohol/medication misuse and domain scores	Greater alcohol use associated with EE and DP
Looseley et al. (72)^*^	Anesthesiologists	397	Prevalence of alcohol intake in burnout groups	No significant difference in alcohol intake
Mikalauskas et al. (84)^*^	Anesthesiologists and intensive care physicians	220	Odds of burnout in those with alcohol abuse/abuse of sedative medications	Alcohol: OR 3.2 (*p* < 0.01) Sedative medication: OR 4.9 (*p* < 0.05)
Oreskovich et al. (83)	Surgeons	7,197	Compared prevalence of alcohol misuse and dependence in those with burnout vs. without	Misuse: 29.6 vs. 25% (*p* < 0.001) Dependence: 34.9 vs. 25% (*p* < 0.001)
Oreskovich et al. (82)	Physicians (all)	7,206	Compared prevalence of burnout in those with alcohol abuse/dependence disorders compared to those without	52.5 vs. 44.7% (*p* < 0.0001)
Pedersen et al. (85)	Physicians (all)	1,841	Odds of alcohol abuse with burnout and each burnout domain	Burnout: OR = 1.86 (*P* < 0.014) EE: OR = 1.89 (*P* < 0.001) DP: OR = 2.23 (*P* < 0.001) PA: OR = 1.66 (P = 0.008)
Rath et al. (86)^*^	Obstetric/gynecology physicians	369	Odds of screening positive for alcohol misuse in those with burnout	OR 2.93 (*p* < 006)
Siu et al. (35)^*^	Physicians (all)	226	Presence of increased alcohol consumption in those with high burnout vs. low burnout	No association with increased alcohol consumption
Talih et al. (54)^*^	Interns and residents (all)	118	Likelihood of increased alcohol consumption and depression	No significant association with alcohol consumption.
Toral-villanueva et al. (80)^*^	Junior doctors	312	Odds of alcohol or drugs misuse in those with burnout	No significant association with alcohol or drugs Alcohol: OR 1.4 (95% CI 0.8–2.5) Drugs: OR 3.0 (95% CI 0.5–16.7)

^*^Assessed multiple outcomes.

### 3.3. Qualitative

#### 3.3.1. Study design and aims

A total of seven qualitative studies were included in the review (87–92, 94). Five studies were purely qualitative in design (87–91) and two used mixed methods (92, 94). All qualitative studies used in depth interviews as a data collection method. Number of participants ranged from 10 (90) to 47 (88). Qualitative study characteristics are outlined in Table 6.

**Table 6:** Qualitative study characteristics.

**References**	**Study design**	**Participants**	**Country**	**Number of participants**	**Research method**	**Study aim**	**Data analysis**
Hamader et al. (87)	Qualitative	Junior doctors	Germany	11	In-depth interview	Analyze reasons and possible interventions for rising levels of burnout, anxiety, depression.	Mayrings method of content analysis
Daruvala et al. (92)	Mixed-methods	Oncology physicians	India	28	In-depth interview	To explore burnout and its associations.	Coding and thematic analysis
Loiselle et al. (94)	Mixed-methods	Academic physicians (all specialties)	USA	40	In-depth interviews	A transcendental meditation technique—randomized control trial. To assess whether technique decreased burnout/depression/anxiety.	Phenomenological analysis
Riley et al. (88)	Qualitative	General practitioners	UK	47	In-depth interview	Reporting experience of GPs living with distress and mental illness.	Coding and thematic analysis
Spiers et al. (89)	Qualitative	General practitioners	UK	47	In depth interviews	Exploring barriers and facilitators to help seeking in GPs with mental distress (depression/anxiety/suicidal ideation and/or burnout).	Coding and thematic analysis
Spiers et al. (90)	Qualitative	General practitioners	UK	10	In depth interviews	Deeper analysis of a small subset of a larger study to understand experience of GPs living with severe mental illness.	Phenomenological analysis
Wainwright et al. (91)	Qualitative	Anesthesia trainees	UK	12	Semi-structured interviews	Identify the personal and professional factors associated with the development of burnout/depression/stress.	Thematic analysis

#### 3.3.2. Qualitative quality assessment

Quality assessment of qualitative studies was conducted using the CASP Tool for qualitative studies (33). The included qualitative studies and their quality appraisal scoring can be seen in Supplementary Table 6. The tool contains ten questions relating to research design, recruitment, data collection including relationship with participants and ethical issues and data analysis and findings. Each question is answered yes, no or unclear as appropriate. For the purposes of this review, studies were given a rating of low, moderate or high based on number of questioned answered yes. Studies were considered high quality if nine questions or more were answered yes, moderate if between five and eight questions were answered yes and low if less than five questions were answered yes.

Three studies were given considered high quality (88, 91, 92), three were considered moderate quality (89, 90, 94) and one study was deemed low quality (87).

##### 3.3.2.1. Study design and recruitment

All included studies outlined the aims and research question clearly and all were suited to qualitative research methods. Recruitment of participants was clearly described and justified by Daruvala et al. (92), Riley et al. (88) and Wainwright et al. (91) including details regarding whether saturation was achieved and how this was decided. Spiers et al. (89) attempted to recruit participants from groups that self-identified as living with mental illness as well as groups living without or recovered, however recruitment for those living with mental illness was much more successful than other groups. There was no mention of recruitment method in other studies (87), introducing the possibility of selection bias.

##### 3.3.2.2. Data collection

All included studies used in-depth interviews as means of data collection. All but one study (87) clearly described interview strategy including whether cues and prompts were used. A sample of topics guides used were provided two studies (88, 89). The relationship between the interviewer and participants was addressed by four studies with only one study specifically stating that reflexivity was practiced by interviewers throughout the process (88). Potential ethical concerns were discussed and addressed by four studies (89–92).

##### 3.3.2.3. Data analysis and findings

Rigorous data analysis was employed by six of the seven included studies (88–92, 94). This included detailed description of coding of themes and the process of analysis, with contribution from multi-disciplinary research team members and sufficient evidence to demonstrate findings.

#### 3.2.3. Data analysis and synthesis

Four studies used a thematic analysis of qualitative data (88, 89, 91, 92), whereby content is analyzed to identify recurring patterns or themes (105). Two studies (90, 94) adopted a phenomenological data analysis method which attempts to analyze the meaning behind the personal experience of a phenomenon described (106). Content analysis was used in one study (87), which focuses on the language of qualitative data and aims to categorize verbal content (107).

#### 3.2.4. Qualitative results

##### 3.2.4.1. Workload, exhaustion, and loss of work-life balance

Work-environment factors featured as an important factor common to the development of both burnout and mental illness in six of the included qualitative studies. Findings from Riley et al. (88) suggest that burnout is most strongly linked to lack of empathy related to chronic overwork, one study participant described how “working too many sessions…you lose your milk of human kindness.” The theme of lack of time for personal life and non-clinical duties including shift duration, lack of leave and night duty is also explored by both Daruvala et al. (92) and Hamader et al. (87). Hamader et al. (87) aimed to highlight reasons for high levels of burnout and depression and ranked lack of sleep, long shifts, stressful shifts and night shifts among their top causes of burnout, anxiety and depression. Similarly, Loiselle et al. (94) and Wainwright et al. (91) document exhaustion due to multiple commitments and lack of time for non-clinical duties as factors in the development of burnout and depression. Nearly all interviewees in Wainwright's (91) study discussed how both work-related and non-clinical work-related pressure led to bouts of exhaustion with one participant describing it as “constantly running on empty.”

##### 3.2.4.2. Chronic workplace stress and interpersonal relationships

Almost all participants in a report on the experience of general practitioners living with mental illness describe chronic states of anxiety at work (88), with some reporting physical symptoms of anxiety such as panic attacks, hyperventilation and nausea. Other participants describe features of depression related to work such as crying on way home from a day's work or easy irritability and anger. Spiers et al. (90), Wainwright et al. (91), and Hamader et al. (87) describe factors identified as contributing to the development of chronic work-related stress. Wainwright et al. (91) identifies a number of participants who spoke about feeling unsupported and unsafe or on-edge at work. Both Hamader et al. (88) and Spiers et al. (90) identify lack of collegiality and unsupportive workplace relationships as a source of work-related stress. One participant used language described as “violent” when describing the interactions with colleagues such as “awful,” “livid,” and “blood bath” (90). However, Spiers et al. (90) and Wainwright et al. (91) also discuss and acknowledge the protective role of supportive work environments and relationships. Wainwright et al. (91) describes the importance of sharing the training experience with peers and Spiers et al. (90) gives numerous examples of how the supportive relationships offer an outlet for inevitable work-related distress.

##### 3.2.4.3. Culture of invulnerability

When investigating barriers and facilitators to help seeking among distressed physicians Spiers et al. (90) identifies a “culture of invulnerability” among physicians, with similar themes described by Wainwright et al. (91), Loiselle et al. (94), and Riley et al. (88). Spiers et al. (90) described the pressure toward “presenteeism” among physicians with several participants describing being ill as a sign of failure. Riley et al. (88) also describes the perception of illness as “failure” or as one interviewee describes it the belief “that you're not strong enough.” Wainwright et al. (91) records how participants perceived others to think that seeking professional help is a sign of weakness. An important example of the normalization of chronic stress and illness is a description of how suicidal thoughts can become both constant and normalized among general practitioners, with one interviewee describing it as “filling his waking thoughts and nights” (88). Loiselle et al. (94) attributes the normalization of chronic stress to lack of knowledge of self-care and concludes that this has implications for managing burnout which ultimately leads to clinical depression.

##### 3.2.3.4. Intervention targets

Three studies discuss possible intervention targets to prevent the development of work-related stress, burnout, depression and anxiety. Similar to the overarching themes, described in all studies, intervention targets broadly relate to work conditions and time spent at work, work relationships and the culture of invulnerability in the workplace. Spiers et al. (90) describes “survival strategies” employed by GPs living and working with mental illness. These include “asserting boundaries” such as not taking on too much and therefore maintaining a work-life balance. Wainwright et al. (91) also highlights the importance of time for non-clinical activities as a target to prevent the development of burnout, anxiety and depression. Both Hamader et al. (87) and Wainwright et al. (91) describe how adequate support, supervision and mentorship may be preventative, by providing psychological support and promoting collegial exchange. Spiers et al. (90) and Wainwright et al. (91) discuss the need to address stigma and move toward a culture change that acknowledges and supports distress at work. Finally, Wainwright et al. (91) highlights a recognition among physicians about their own responsibility for self-care, an important target for managing chronic occupational stress.

## 4. Discussion

### 4.1. Integration of qualitative and quantitative findings

Quantitative findings suggest significant relationships between physician burnout and depression and anxiety but less significant or unclear relationships between physician burnout and substance abuse and suicidality. Qualitative studies explored factors related to burnout, anxiety, depression and suicidality as a continuum and add insight and depth to the association identified in quantitative studies. Key areas explored that relate to the development and progression of both exposure and outcome included work related factors such as daily stress and time pressure, challenging or supportive workplace relationships and culture regarding sick leave, mental illness and help-seeking. This will be explored further in the discussion section.

Daily work-related stress exacerbated by the quantity of time spent at work and the lack of time for personal activities formed a central theme of qualitative data. Many quantitative studies also explored and addressed factors described in qualitative interviews and their mediating roles in the development and progression of burnout. Williford et al. (55), who found a statistically significant increase in depression scores in those with burnout asked participants to rank factors that they perceived to be associated with the risk of developing burnout. The highest ranked factor was lack of time for exercise, self-care and doing things they enjoyed. This was followed by conflicting work and personal commitments, a by-product of lack of time. While several quantitative studies investigated the association between hours worked, shift work and burnout or outcomes, results were inconsistent. Nomura et al. (93) and Haik et al. (59) found no significant association between hours worked, night shifts and burnout and Shanafelt et al. (65) similarly reported no association between hours worked and suicidal ideation. There were however frequent associations drawn between outside work activities and decreased levels of burnout and depression, such as time for self-care (92) and increased levels of physical activity (47). This might suggest that number of hours worked or shift patterns may be less important than quality of time spent not at work and work-life balance. Janko et al. (62) found higher levels of burnout in those without access to programmatic social events. Wainwright et al. (91) suggests that recognition of the importance of self-care such as prioritizing outside work activities may be an important target for the prevention of burnout, depression and stress.

The role of collegiality and work relationships is another factor that qualitative studies suggest links workplace stress with burnout and progression to mental illness. While describing the experience of physicians living with mental illness, Spiers et al. (89) describes the influence of poor work relationships as “sometimes actively destructive” but also highlights the protective effect of collegial relationships. Supportive mentorship is suggested as an outlet for the inevitable work-related stress experienced by general practitioners. Hyman et al. (60), who found significant correlation between mental composite scores and burnout scores, also found that professional and personal support was associated with lower EE scores. Janko et al. (62) also identified that those in the highest burnout quartile had higher rates of depression also found that trainees with a self-identified mentor had significantly lower overall burnout scores.

Finally, a culture of invulnerability among physicians is described by both Hamadar et al. (87) and Riley et al. (88) whereby admitting personal struggle and accessing support services is seen as “weakness” or “failure.” Riley et al. (87) noted that although feelings of chronic anxiety were prevalent among General Practitioners, even some of those affected did not identify as having mental ill-health. One large cross-sectional study that found a significant association between burnout and suicidal ideation that was independent of depression and symptoms of depression also found that those with suicidal ideation were less likely to seek professional help and more likely to self-prescribe (65). Talih et al. (54) reported a positive association between burnout, depression, anxiety and suicidal ideation and also found that burnout correlated with self-administration of psychotropic medication and that more than 50% of those with suicidal ideation had not sought professional help. Reluctance to seek professional help was also reported to be associated with burnout by Rath et al. (86) as well as with depression and substance abuse. Willford et al. (55) asked study participants to rank barriers to help seeking in severe burnout, shame and denial ranked among the highest reasons for not accessing support services. Spiers et al. (89) noted that no participant spontaneously spoke about available support services despite describing personal issues with burnout and mental health.

None of the included qualitative studies explored alcohol or substance abuse as an outcome of burnout or occupational stress. This adds weight to the inconsistent findings of quantitative studies, suggesting that substance or alcohol abuse may be a coping mechanism that is more individual specific instead of directly correlated.

### 4.2. Findings in the context of existing literature

A review of burnout literature that analyzed quantity and content of publications prior to 2011 described an exponential rise in relevant material after 2005 (28). Considering that studies included in the current systematic review were predominantly published after this date, it is apparent that its relevance continues to increase. The large volume of recently published material prompts the need for methodologically appropriate assimilation and interpretation of data. While a number of systematic reviews of the literature have been published, the focus primarily has been on prevalence in different subgroups, interventions, and the impact on patient centered outcomes such as patient safety and quality of healthcare delivery. To the best of our knowledge this is the only systematic review to investigate the relationship between physician burnout and the specific outcomes of depression, anxiety, suicidality and substance abuse and to include qualitative findings to explore the perceived links between exposure and outcome.

The results of this review indicate a consistently positive and strong association between burnout and depression and between burnout and anxiety. This is in keeping with findings among other occupations. Koutsimani et al. (108) reported similar findings in a systematic review that examined the relationship between burnout and depression and burnout and anxiety in all employed adults. The review included only studies that measured correlation and performed a meta-analysis. It was noted that the correlation was not so strong as to suggest that burnout, depression or anxiety are the same entity and concludes that treating them as separate constructs will have implications for potential intervention and targeted solutions. There was also significant heterogeneity of included studies in the review, for reasons similar to those that precluded meta-analysis in the current review.

The current systematic review found inconsistent association between burnout and suicidality and between burnout and substance abuse. Suicide has a well-documented association with depression, however despite similar prevalence of depression among physicians and the general population (109) rates of suicide are higher (110). The question remains as to whether burnout acts a predictor of suicidality independent of depression. Shanafelt et al. (65) reported a strong association between burnout and suicidal ideation that increases with burnout severity and argues that burnout is an independent risk factor after controlling for depression. Medical students were excluded from the current review however a large cohort study of American medical students also found burnout to be independent risk factor for suicidal thoughts and reports reversibility of suicidal ideation with recovery from burnout over time (109). Inconsistencies in results regarding suicidality may be attributed to the challenges measuring suicidal ideation or difference between suicidal ideation and overall suicide behavior. Suicidal ideation is one of the strongest predictors for suicidal acts (111) but is subjective and notoriously difficult to measure (112). Both stigma and “normalization” of suicidal thoughts, as described in qualitative studies included in the review, may lead to study participants denying true intentions. A study that investigated whether the suicidal ideation section of the PHQ 9 predicted suicide in American Veterans found that suicidal ideation as indicated by response to PHQ 9 was significantly associated with death by suicide. It was also reported however, that 71% of suicides that occurred during the study time period occurred among those who reported no suicidal ideation (113).

Findings relating to alcohol and substance misuse favor no or very small association with burnout with the majority of studies finding no significant association. Four studies did find increased rates of alcohol misuse in those with burnout indicating that an association may exist. Pederson et al. (85) noted a significant association that was strongest in the DP domain. DP has been discussed in the literature as a coping strategy for EE (114). It may follow that substance and alcohol abuse may be a maladaptive coping mechanism employed by some physicians with burnout instead of directly and uniformly correlated. It is likely that genetic predisposition, opportunity and personal factors also contribute (115). A qualitative analysis of physicians abusing prescription medication found that those using substances to alleviate stress and anxiety had initially been using the medication to manage pain and were self-prescribing, indicating that substance misuse did not necessarily directly result from stress but may occur as a form of self-treatment (116). Measurement of substance and alcohol misuse present similar challenges as often those effected will be reluctant to admit to the problem for fear of repercussion.

Only seven qualitative studies that addressed the research question were identified by the current review indicating a relative scarcity of qualitative data. However, qualitative research surrounding burnout as a phenomenon does exist. A systematic review and qualitative metasynthesis of physician's perspectives on burnout describes organizational, relation and individual factors associated with the development of burnout (117). The review ranks and structures the factors as a timeline. The development of burnout is described as beginning with organizational factors such high workload, stress and lack of time, followed by relational factors including relational difficulties with other professionals and ultimately individual factors such as guilt, helplessness and doubt are experienced last. Factors highlighted as protective mirror stress factors but are ranked in the opposite direction. Individual protective factors such as self-care were considered most important followed by supportive relationships and finally organizational factors. The researchers point out that those affected will protect themselves individually first and foremost but suggest that relation and organization protective factors should play a more prominent role in the prevention of burnout, given their role in its development. Although this qualitative analysis of burnout contains no mention of other psychological outcomes, many of the same themes are explored in relation to the progression of burnout and development of mental illness in the current review. This would suggest that the factors identified as risk factors for the development of burnout may be implicated in the development of depression, anxiety or suicidal ideation without intervention.

Qualitative studies included in the current review explore both a “culture of invulnerability” that exists among physicians and “normalization” of chronic stress, anxiety and features of mental illness including suicidal thoughts. These factors are explored as barriers to help seeking that may contribute to the progression of burnout to psychological morbidity. This may also offer insight into the reason for the timeline of protective factors described by Sibeoni et al. (117). Admitting to personal struggle has been described as “failure” or “weakness” thus prompting physicians to seek individual solutions such as resilience and self-care first. Lebares et al. (118) found that mindfulness and resilience traits were associated with lower levels of burnout, anxiety and depression but also suggests that organizational based interventions should be developed concomitantly. Fostering a healthy work environment would support the individual while allowing them to draw on their natural strengths. This is supported by two systematic reviews designed to investigate and compare burnout interventions that suggest that organizational based interventions have a higher treatment effect (119) which is longer lasting (120) than individual based interventions.

In a systematic review that examines the personal and professional consequences of burnout in physicians, Williams et al. (121) describes the phenomenon of burnout as a “loss spiral” or “burnout cascade.” It is hypothesized that the severity of consequence relates to the progression of burnout. Initially loss of empathy or intention to leave predominate, followed by depression or anxiety and finally suicidal ideation or physical health problems at the terminal end of the spectrum. Burnout is considered as a continuum rather than an end-state and research attesting to positive association between burnout and all factors considered to be relevant to the “loss spiral” is explored. This study most closely relates to the current review and findings complement one another. Both report significant association between burnout and depression and burnout and anxiety. These outcomes are studied in more detail in the current review with inclusion of more relevant studies adding weight and credibility to the association. The current review finds a weaker association between burnout and suicidality which may be accounted for by the association only occurring in the case of severe or end-stage burnout as is described by Williams et al. (116). Williams et al. (116) reports an association with alcohol abuse and it is described as a means of coping with increasing distress. The current review questions this association as results are inconsistent and favor no direct association. However, as discussed, individual coping style and personal factors may account for the link between burnout and alcohol abuse. Williams et al. (116) discuss the need for organizational processes and effective leadership in preventing progression through the burnout cascade through targeted interventions. Changing the workplace culture that discourages help-seeking and promoting team-work and support are described as “resources” that make up for the loss in the “loss spiral.” Qualitative findings from the current review that explore workplace stress, burnout, depression, anxiety and suicidality as continuum support this view.

### 4.3. Limitations

There was limitation from the review methods and search strategy chosen as due to language and budgetary constraints, studies with no English translation were excluded allowing for potential exclusion of relevant studies.

There is considerable heterogeneity of included studies particularly relating to population characteristics and measurement of exposure and outcome. This not only precludes meta-analysis of results but may also affect the generalizability of findings. Most studies featured subgroups of physicians and focused on either one institution or one group of institutions. Although study results are not sufficiently different as to suggest that degree of association was significantly different between subgroups, caution must be taken when considering study results together. There were also notable differences in method of measurement of exposure and interpretation of measurement results. Although the most commonly used measurement tool was used in 46 of 54 studies, interpretation of measurement score was much more variable. While 17 studies evaluated burnout domain scores as separate continuous variables as is recommended by MBI guidelines (114) the remainder used alternative interpretations such as defining burnout “cutoff” scores and evaluating burnout as a dichotomous variable. While rationale for interpretation is clearly outlined in all studies, differences may influence overall strength of association as the interpretation of exposure is not consistent across all studies. Previous systematic reviews have noted differences in effect sizes when different measurement tools are used and specifically noted lower effect sizes when the MBI is used compared to other burnout measurement tools (122). Results may therefore differ depending on measurement tool used and may distort results when considered as a whole.

This review was carried out prior to the increase in burnout literature relating to physicians and burnout related to the COVID pandemic. Future studies could update this review encompassing evolving literature studying the association of depression, anxiety, substance abuse and suicidality within the context of workplace burnout during and after the COVID Pandemic.

There are a number of limitations within the included studies which need to be considered. For example the participants of included studies may have been subject to selection bias and not representative of the study population. By design cross-sectional analysis is vulnerable to selection bias (123). Participation was voluntary across all studies and therefore respondents may represent a self-selected group. Specific to this review, participants with burnout may be less likely to respond due to lack of time or motivation or alternatively more likely to respond due to interest in the subject matter. This can be addressed with sample size calculation or high response rates. Many studies used internet-based surveys to address confidentiality concerns and encourage response rates. Recall bias can play a role in the measurement of exposure and outcome. Cross-sectional analysis takes place in a single moment in time and therefore responses may only be representative of current state or short time-period prior to survey as opposed to overall long-term state. Further to this cross-sectional analysis allows for limited means to account for confounding factors may distort reported association between exposure and outcome (123). Only half of included studies addressed the possibility of confounding. One study used a control group to control for confounding; however, this study was deemed to have low overall quality.

Additionally, often there was inability of study design to determine causality. Many studies report the depression or depressive symptoms as a risk factor for burnout calling into question the direction of causality.

### 4.4. Recommendations

Recommendations:

- The current review not only highlights the volume of studies that address the research question but also gives an indication as to the gaps that continue to exist in the literature. The inability of cross-sectional studies to provide information regarding the temporal relationship between exposure and outcome is the most frequently discussed limitation among included quantitative studies. The consistency of significant findings regarding the association between burnout and depression and burnout and anxiety suggest that an association does exist however it is impossible, based on study design, to make any assumption regarding causality or direction of association. A well-designed prospective cohort study could give insight into how burnout develops over time, how severity changes in the face of different factors and whether other psychological outcomes precede, coincide with or occur as a result of burnout.
- The relative scarcity of qualitative literature compared to quantitative studies suggests the need for further qualitative investigation of the research question. Literature suggests that burnout is a complex process that does not occur in a vacuum but encompasses multiple social, environmental and personal facets. Full exploration of such a phenomenon benefits from in-depth qualitative analysis that can add insight, detail and context to findings measured by quantitative analysis.
- Meaningful assimilation and comparison of study results is limited by the heterogeneity of burnout measurement, most notably the lack of consensus regarding the interpretation of burnout measurement scores and importance of burnout domains. Future research would benefit from clarity regarding the definition of burnout and burnout domains.
- Despite the discussed limitations and gaps in knowledge provided by the current review, findings allow a number of suggestions for practice and policy. Results regarding physician burnout and depression are consistent and thus highlight the importance of the relationship. Although a smaller number, findings are similarly consistent for burnout and anxiety. This identifies burnout as a possible target for intervention to prevent more serious psychological illness indicating the importance of its early recognition. Qualitative data provides detailed description of how feelings of depression and anxiety relate to workplace stress and highlights perceived links. Although intervention is outside the scope of this review the results highlight how intervention may be considered.
- Qualitative data suggests that the relationship is influenced by both individual factors such as work-life balance and organizational factors such as collegiality and the culture and stigma surrounding wellbeing and help-seeking. This suggests that both individual and organizational interventions are required to fully address the problem of burnout and its progression. A recent review of interventions targeting physician burnout described primarily interventions relating to individual factors such as relaxation techniques and coping strategies (124). Our review would suggest that equal emphasis should be placed on organizational interventions that promote collegiality and a workplace culture that acknowledges vulnerability and encourages help-seeking.

## 5. Conclusion

The current systematic review presents findings suggestive of a significant association between both burnout and depression and burnout and anxiety in physicians and an important relationship between burnout and suicidality. The relationship between substance misuse and physician burnout is less clear with results indicating that any association may be related to specific components of burnout or confounded by other personality or environmental variables. Lack of longitudinal data limits any assumption regarding causality or direction of the association and is therefore a suggested target for future research. Similarly, heterogeneity of criteria used to define burnout limits comparison of results and future research may benefit from consensus regarding burnout measurement. Detailed description of the manifestations of chronic workplace stress, burnout, depression, anxiety and suicidal ideation provided by qualitative results highlighting the importance, nature and consequences of the relationship. Qualitative data also suggests perceived links that facilitate the progression of workplace stress and burnout to psychological outcomes including lack of time for work-life balance, a workplace culture that normalizes psychological distress acting as a barrier to help-seeking and poor collegial relationships. The WHO defines burnout as a problem related to employment (3). Our results indicate that as such it may act as risk factor for more serious psychological morbidity in physicians and that both individual and organizational interventions may be beneficial.

## Data availability statement

The original contributions presented in the study are included in the article/Supplementary material, further inquiries can be directed to the corresponding author.

## Author contributions

ER conceived the study idea and conducted data analysis. ER and TJ contributed to study design. ER and KH conducted the search, screening, and data extraction. ER, TJ, and JP drafted the manuscript. All authors approved the manuscript for publication.

## References

[1] Maslach C Jackson SE. Burned-out cops and their families. Psychol Today. (1979) 12:59–62.24160171

[2] Freudenberger HJ. Staff burn-out. J Soc Issues. (1974) 30:159–65. 10.1111/j.1540-4560.1974.tb00706.x

[3] The World Health Organisation. WHO | International Classification of Diseases, 11th Revision (ICD-11). Internation Classification of Diseases 11th Revision (ICD-11) (2019).

[4] Afonso AM Cadwell JB Staffa SJ Zurakowski D Vinson AE. Burnout rate and risk factors among anesthesiologists in the United States. Anesthesiology. (2021) 134:683–96. 10.1097/ALN.0000000000003722 33667293 PMC9430843

[5] Jun J Ojemeni MM Kalamani R Tong J Crecelius ML. Relationship between nurse burnout, patient and organizational outcomes: systematic review. Int J Nurs Stud. (2021) 119:103933. 10.1016/j.ijnurstu.2021.103933 33901940

[6] Center C Davis M Detre T Ford DE Hansbrough W Hendin H . Confronting depression and suicide in physicians: a consensus statement. JAMA. (2003) 289:3161–6. 10.1001/jama.289.23.3161 12813122

[7] Frank E Biola H Burnett CA. Mortality rates and causes among U.S. physicians. Am J Prev Med. (2000) 19:155–9. 10.1016/S0749-3797(00)00201-4 11020591

[8] Rotenstein LS Torre M Ramos MA Rosales RC Guille C Sen S . Prevalence of burnout among physicians a systematic review. JAMA. (2018) 320:1131–50. 10.1001/jama.2018.12777 30326495 PMC6233645

[9] Rathert C Williams ES Linhart H. Evidence for the quadruple aim: a systematic review of the literature on physician burnout and patient outcomes. Med Care. (2018) 56:976–84. 10.1097/MLR.0000000000000999 30339573

[10] Dewa CS Loong D Bonato S Thanh NX Jacobs P. How does burnout affect physician productivity? A systematic literature review. BMC Health Serv Res. (2014) 14:325. 10.1186/1472-6963-14-325 25066375 PMC4119057

[11] Han TD S Shanafelt Sinsky CA Awad KM Dyrbye LN Fiscus LC . Estimating the attributable cost of physician burnout in the United States. Ann Intern Med. (2019) 170:784–90. 10.7326/M18-1422 31132791

[12] Maslach C Leiter MP. Understanding the burnout experience: recent research and its implications for psychiatry. World Psychiatry. (2016) 15:103. 10.1002/wps.20311 27265691 PMC4911781

[13] Carpenter LM Swerdlow AJ Fear NT. Mortality of doctors in different specialties: findings from a cohort of 20 000 NHS hospital consultants. Occup Environ Med. (1997) 54:388–95. 10.1136/oem.54.6.388 9245944 PMC1128798

[14] Schernhammer ES Colditz GA. Suicide rates among physicians: a quantitative and gender assessment (meta-analysis). Am J Psychiatry. (2004) 161:2295–302. 10.1176/appi.ajp.161.12.2295 15569903

[15] Sansone RA Sansone LA. Physician suicide: a fleeting moment of despair. Psychiatry. (2009) 6:18. 19724738 PMC2719447

[16] Brooks SK Gerada C Chalder T. Review of literature on the mental health of doctors: are specialist services needed? J Mental Health. (2011) 20:146–56. 10.3109/09638237.2010.541300 21275504

[17] Tyssen R Vaglum P Gronvold NT Ekeberg O NT G Ekeberg Ø. The impact of job stress and working conditions on mental health problems among junior house officers. A nationwide Norwegian prospective cohort study. Med Educ. (2000) 34:374–84. 10.1046/j.1365-2923.2000.00540.x 10760123

[18] Alonso J Angermeyer MC Bernert S Bruffaerts R Brugha TS Bryson H . Prevalence of mental disorders in Europe: results from the European Study of the Epidemiology of Mental Disorders (ESEMeD) project. Acta Psychiatr Scand. (2004) 109:21–7. 10.1111/j.1600-0047.2004.00325.x 15128384

[19] Rose GL Brown RE. The impaired anesthesiologist: not just about drugs and alcohol anymore. J Clin Anesth. (2010) 22:379–84. 10.1016/j.jclinane.2009.09.009 20650388

[20] Swendsen JD Merikangas KR. The comorbidity of depression and substance use disorders. Clin Psychol Rev. (2000) 20:173–89. 10.1016/S0272-7358(99)00026-4 10721496

[21] Marshall EJ. Doctors' health and fitness to practise: treating addicted doctors. Occup Med. (2008) 58:334–40. 10.1093/occmed/kqn081 18676427

[22] Morse RM Martin MA Swenson WM Niven RG. Prognosis of physicians treated for alcoholism and drug dependence. JAMA. (1984) 251:743–6. 10.1001/jama.251.6.743 6694277

[23] Bosch X. First impaired physicians therapy program appears to be successful in Spain. J Am Med Assoc. (2000) 283:3186–7. 10.1001/jama.283.24.3186-JMN0628-2-1 10866848

[24] Renzi C Di Pietro C Tabolli S. Psychiatric morbidity and emotional exhaustion among hospital physicians and nurses: association with perceived job-related factors. Arch Environ Occup Heal. (2012) 67:117–23. 10.1080/19338244.2011.578682 22524653

[25] Vandevala T Pavey L Chelidoni O Chang N-F Creagh-Brown B Cox A. Psychological rumination and recovery from work in intensive care professionals: associations with stress, burnout, depression and health. J Intensive Care. (2017) 5:16. 10.1186/s40560-017-0209-0 28174662 PMC5290656

[26] Bianchi R Boffy C Hingray C Truchot D Laurent E. Comparative symptomatology of burnout and depression. J Health Psychol. (2013) 18:782–7. 10.1177/1359105313481079 23520355

[27] Messias E Flynn V. The tired, retired, and recovered physician: professional burnout versus major depressive disorder. Am J Psychiatry. (2018) 175:716–9. 10.1176/appi.ajp.2018.17121325 30064240

[28] Heinemann LV Heinemann T. Burnout research: emergence and scientific investigation of a contested diagnosis. SAGE Open. (2017) 7:2158244017697154. 10.1177/2158244017697154

[29] Moher D Liberati A Tetzlaff J Altman DG Group TP. Preferred reporting items for systematic reviews and meta-analyses: the PRISMA statement. PLoS Med. (2009) 6:e1000097. 10.1371/journal.pmed.1000097 19621072 PMC2707599

[30] Severinsson E. Moral stress and burnout: qualitative content analysis. Nurs Heal Sci. (2003) 5:59–66. 10.1046/j.1442-2018.2003.00135.x 12603722

[31] Munn Z Stern C Aromataris E Lockwood C Jordan Z. What kind of systematic review should i conduct? A proposed typology and guidance for systematic reviewers in the medical and health sciences. BMC Med Res Methodol. (2018) 18:5. 10.1186/s12874-017-0468-4 29316881 PMC5761190

[32] Joanna Briggs Institute. Critical Appraisal Tools. (2005). Vol. 6. p. 2006. Available online at: http://www.cebm.net/critical-appraisal/

[33] Critical Appraisal Skills Programme. CASP Qualitative Checklist. Casp (2018). p. 1. Available online at: http://www.casp-uk.net/casp-tools-checklists (accessed January 21, 2022).

[34] Pearson A White H Bath-Hextall F Salmond S Apostolo J Kirkpatrick P. A mixed-methods approach to systematic reviews. Int J Evid Based Healthc. (2015) 13:121–31. 10.1097/XEB.0000000000000052 26196082

[35] Siu Dr. CFY, Yuen SK, Cheung Dr. A. Burnout among public doctors in Hong Kong: cross-sectional survey. Hong Kong Med J. (2012) 18:186–92. 22665681

[36] Campbell J Prochazka A V Yamashita T Gopal R. Predictors of persistent burnout in internal medicine residents: a prospective cohort study. Acad Med J Assoc Am Med Coll. (2010) 85:1630–4. 10.1097/ACM.0b013e3181f0c4e7 20881685

[37] Carter RD. Physician wellness: impact of stress, burnout, and depression on medical trainee empathy.*Dissert Abstracts Int Sect A Hum Soc Sci*. (2019) 80.32482121

[38] Chaukos D Chad-Friedman E Mehta DH Byerly L Celik A McCoy THJ . Risk and resilience factors associated with resident burnout. Acad Psychiatry. (2017) 41:189–94. 10.1007/s40596-016-0628-6 28028738

[39] Van der Heijden F Dillingh G Bakker A Prins J van der Heijden F Dillingh G . Suicidal thoughts among medical residents with burnout. Arch Suicide Res Off J Int Acad Suicide Res. (2008) 12:344–6. 10.1080/13811110802325349 18828037

[40] Faivre G Kielwasser H Bourgeois M Panouilleres M Loisel F Obert L. Burnout syndrome in orthopaedic and trauma surgery residents in France: a nationwide survey. Orthop Traumatol Surg Res. (2018) 104:1291–5. 10.1016/j.otsr.2018.08.016 30341030

[41] Ferrari S Cuoghi G Mattei G Carra E Volpe U Jovanovic N . Young and burnt? Italian contribution to the international BurnOut Syndrome Study (BOSS) among residents in psychiatry. Med Lav. (2015) 106:172–85. 25951864

[42] Govardhan LM Pinelli V Schnatz PF. Burnout, depression and job satisfaction in obstetrics and gynecology residents. Conn Med. (2012) 76:389–95. 23248861

[43] Juntunen J Asp S Olkinuora M Aärimaa M Strid L Kauttu K. Doctors' drinking habits and consumption of alcohol. BMJ. (1988) 297:951–4. 10.1136/bmj.297.6654.951 3142564 PMC1834675

[44] Karaoglu N Pekcan S Durduran Y Mergen H Odabasi D Ors R. A sample of paediatric residents' loneliness-anxiety-depression-burnout and job satisfaction with probable affecting factors. J Pak Med Assoc. (2015) 65:183–91. 25842556

[45] Lazarescu I Dubray B Joulakian MB Blanchard P Chauvet B Mahé M-A . Prevalence of burnout, depression and job satisfaction among French senior and resident radiation oncologists. Cancer Radiother J La Soc Fr Radiother Oncol. (2018) 22:784–9. 10.1016/j.canrad.2018.02.005 30348608

[46] Lebares CC Guvva E V Ascher NL O'Sullivan PS Harris HW Epel ES . Burnout and stress among US surgery residents: psychological distress and resilience. J Am Coll Surg. (2018) 226:80–90. 10.1016/j.jamcollsurg.2017.10.010 29107117

[47] Lebensohn P Dodds S Benn R Brooks AJ Birch M Cook P . Resident wellness behaviors: relationship to stress, depression, and burnout. Fam Med. (2013) 45:541–9. 24129866

[48] Lu DW Dresden S McCloskey C Branzetti J Gisondi MA Branzetti J. Impact of burnout on self-reported patient care among emergency physicians. West J Emerg Med. (2015) 16:996–1001. 10.5811/westjem.2015.9.27945 26759643 PMC4703144

[49] Mohammed KA-M Ali EG Youssef IM Fahmy MT Haggag WE. Depression and burnout among residents. Arab J Psychiatry. (2014) 25:40–51. 10.12816/0004114

[50] Nishimura Y Miyoshi T Obika M Ogawa H Kataoka H Otsuka F. Factors related to burnout in resident physicians in Japan. Int J Med Educ. (2019) 10:129–35. 10.5116/ijme.5caf.53ad 31272084 PMC6766397

[51] Ofei-Dodoo S Kellerman R Gilchrist K Casey EM. Burnout and quality of life among active member physicians of the medical society of Sedgwick County. Kansas J Med. (2019) 12:33–9. 10.17161/kjm.v12i2.11701 31191807 PMC6527197

[52] Pasqualucci PL Damaso LLM Danila AH Fatori D Lotufo Neto F Koch VHK. Prevalence and correlates of depression, anxiety, and stress in medical residents of a Brazilian academic health system. BMC Med Educ. (2019) 19:193. 10.1186/s12909-019-1621-z 31185960 PMC6558838

[53] Sun W Fu J Chang Y Wang L. Epidemiological study on risk factors for anxiety disorder among chinese doctors. J Occup Health. (2012) 54:1–8. 10.1539/joh.11-0169-OA 22156318

[54] Talih F Warakian R Ajaltouni J Shehab AAS Tamim H Shehab AAS. Correlates of depression and burnout among residents in a lebanese academic medical center: a cross-sectional study. Acad Psychiatry. (2016) 40:38–45. 10.1007/s40596-015-0400-3 26246222

[55] Williford ML Scarlet S Meyers MO Luckett DJ Fine JP Goettler CE . Multiple-institution comparison of resident and faculty perceptions of burnout and depression during surgical training. JAMA Surg. (2018) 153:705–11. 10.1001/jamasurg.2018.0974 29800976 PMC6584717

[56] Ashraf F Ahmad H Shakeel M Aftab S Masood A. Mental health problems and psychological burnout in Medical Health Practitioners: a study of associations and triadic comorbidity. Pakistan J Med Sci. (2019) 35:1558–64. 10.12669/pjms.35.6.444 31777493 PMC6861496

[57] Boo YL Liam CCK Lim SY Look ML Tan MH Ching SM . Stress and burnout syndrome in health-care providers treating dengue infection: a cross-sectional study. Med J Malaysia. (2018) 73:371–5. 30647206

[58] Bourne T Shah H Falconieri N Timmerman D Lees C Wright A . Burnout, well-being and defensive medical practice among obstetricians and gynaecologists in the UK: cross-sectional survey study. BMJ Open. (2019) 9:e030968. 10.1136/bmjopen-2019-030968 31767585 PMC6887071

[59] Haik J Brown S Liran A Visentin D Sokolov A Zilinsky I . Burnout and compassion fatigue: prevalence and associations among Israeli burn clinicians. Neuropsychiatr Dis Treat. (2017) 13:1533–40. 10.2147/NDT.S133181 28670122 PMC5478274

[60] Hyman SA Shotwell MS Michaels DR Han X Card EB Morse JL . A survey evaluating burnout, health status, depression, reported alcohol and substance use, and social support of anesthesiologists. Anesth Analg. (2017) 125:2009–18. 10.1213/ANE.0000000000002298 28991114

[61] Korkeila J Töyry S Kumpulainen K Toivola J-M Räsänen K Kalimo R . Burnout and self-perceived health among Finnish psychiatrists and child psychiatrists: a national survey. Scand J Public Health. (2003) 31:85–91. 10.1080/14034940210133880 12745757

[62] Janko MR Smeds MR. Burnout, depression, perceived stress, and self-efficacy in vascular surgery trainees. J Vasc Surg. (2019) 69:1233–42. 10.1016/j.jvs.2018.07.034 30301691

[63] Khan A Teoh KR Islam S Hassard J. Psychosocial work characteristics, burnout, psychological morbidity symptoms and early retirement intentions: a cross-sectional study of NHS consultants in the UK. BMJ Open. (2018) 8:e018720. 10.1136/bmjopen-2017-018720 30037857 PMC6059335

[64] Mampuya WA Matsuo Y Nakamura A Hiraoka M. Evaluation of the prevalence of burnout and psychological morbidity among radiation oncologist members of the Kyoto Radiation Oncology Study Group (KROSG). J Radiat Res. (2017) 58:217–24. 10.1093/jrr/rrw094 28399575 PMC5571610

[65] Shanafelt TD Balch CM Dyrbye L Bechamps G Russell T Satele D . Special report: suicidal ideation among American surgeons. Arch Surg. (2011) 146:54–62. 10.1001/archsurg.2010.292 21242446

[66] Thommasen H V Connelly I Lavanchy M Berkowitz J Grzybowski S. Short report: burnout, depression, and moving away. How are they related? Can Fam Physician. (2001) 47:747–9. 11340755 PMC2018429

[67] Wurm W Vogel K Holl A Ebner C Bayer D Mörkl S . Depression-burnout overlap in physicians. PLoS ONE. (2016) 11:e0149913. 10.1371/journal.pone.0149913 26930395 PMC4773131

[68] Yilmaz A. Burnout, job satisfaction, and anxiety-depression among family physicians: a cross-sectional study. J Fam Med Prim Care. (2018) 7:952–6. 10.4103/jfmpc.jfmpc_59_18 30598939 PMC6259531

[69] Zhang Y Chu X Sha Y Zeng X Shen T. Survey of job burnout and depression in standardized residency training programs in China. Medicine. (2019) 98:e16890. 10.1097/MD.0000000000016890 31464919 PMC6736065

[70] Zhou J Yang Y Qiu X Yang X Pan H Ban B . Relationship between anxiety and burnout among Chinese physicians: a moderated mediation model. PLoS ONE. (2016) 11:e0157013. 10.1371/journal.pone.0157013 27479002 PMC4968847

[71] Faivre G Marillier G Nallet J Nezelof S Clment I Obert L. Are French orthopedic and trauma surgeons affected by burnout? Results of a nationwide survey. Orthop Traumatol Res. (2019) 105:395–9. 10.1016/j.otsr.2018.12.009 30819660

[72] Looseley A Wainwright E Cook TM Bell V Hoskins S O'Connor M . Stress, burnout, depression and work satisfaction among UK anaesthetic trainees; a quantitative analysis of the Satisfaction and Wellbeing in Anaesthetic Training study. Anaesthesia. (2019) 74:1231–9. 10.1111/anae.14681 31090924

[73] Whitley TW Gallery ME Allison EJ Jr Revicki DA. Factors associated with stress among emergency medicine residents. Ann Emerg Med. (1989) 18:1157–61. 10.1016/S0196-0644(89)80051-4 2817559

[74] Becker JL Milad MP Klock SC. Burnout, depression, and career satisfaction: cross-sectional study of obstetrics and gynecology residents. Am J Obstet Gynecol. (2006) 195:1444–9. 10.1016/j.ajog.2006.06.075 17074551

[75] Bernburg M Vitzthum K Groneberg DA Mache S. Physicians' occupational stress, depressive symptoms and work ability in relation to their working environment: a cross-sectional study of differences among medical residents with various specialties working in German hospitals. BMJ Open. (2016) 6:e011369. 10.1136/bmjopen-2016-011369 27311909 PMC4916614

[76] Iorga M Socolov V Muraru D Dirtu C Soponaru C Ilea C . Factors influencing burnout syndrome in obstetrics and gynecology physicians. Biomed Res Int. (2017) 2017:1–10. 10.1155/2017/9318534 29359161 PMC5735583

[77] Pompili M Innamorati M Narciso V Kotzalidis GD Dominici G Talamo A . Burnout, hopelessness and suicide risk in medical doctors. Clin Ter. (2010) 161:511–4. 21181078

[78] Sahin B Musaoglu E Dogan B Yildirim A Arslan T Sahin H. Profile differences of medical doctors from three different hospitals in Turkey concerning burnout, job satisfaction, and depression. Klin Psikiyatr Dergisi-Turkish J Clin Psychiatry. (2019) 22:148–56. 10.5505/kpd.2019.73792

[79] Tateno M Jovanović N Beezhold J Uehara-Aoyama K Umene-Nakano W Nakamae T . Suicidal ideation and burnout among psychiatric trainees in Japan. Early Interv Psychiatry. (2018) 12:935–7. 10.1111/eip.12466 28786526

[80] Toral-Villanueva R Aguilar-Madrid G Juárez-Pérez CA. Burnout and patient care in junior doctors in Mexico City. Occup Med. (2009) 59:8–13. 10.1093/occmed/kqn122 18796698

[81] Williamson K Lank PM Cheema N Hartman N Lovell EO. Comparing the maslach burnout inventory to other well-being instruments in emergency medicine residents. J Grad Med Educ. (2018) 10:532–6. 10.4300/JGME-D-18-00155.1 30386478 PMC6194874

[82] Oreskovich MR Shanafelt T Dyrbye LN Tan L Sotile W Satele D . The prevalence of substance use disorders in American physicians. Am J Addict. (2015) 24:30–8. 10.1111/ajad.12173 25823633

[83] Oreskovich MR Kaups KL Balch CM Hanks JB Satele D Sloan J . Prevalence of alcohol use disorders among American surgeons. Arch Surg. (2012) 147:168–74. 10.1001/archsurg.2011.1481 22351913

[84] Mikalauskas A Benetis R Širvinskas E Andrejaitiene J Kinduris Š Macas A . Burnout among anesthetists and intensive care physicians. Open Med. (2018) 13:105–12. 10.1515/med-2018-0017 29666844 PMC5900415

[85] Pedersen AF Sørensen JK Bruun NH Christensen B Vedsted P. Risky alcohol use in Danish physicians: associated with alexithymia and burnout? Drug Alcohol Depend. (2016) 160:119–26. 10.1016/j.drugalcdep.2015.12.038 26832935

[86] Rath KS Huffman LB Phillips GS Carpenter KM Fowler JM. Burnout and associated factors among members of the Society of Gynecologic Oncology. Am J Obstet Gynecol. (2015) 213:824.e1–9. 10.1016/j.ajog.2015.07.036 26226551

[87] Hamader G Noehammer E. Prevention of anxiety, depression and burnout during medical studies and residency training (experts' opinion, medical students' and young doctors' point of view). In:Noehammer E, editor. Psychology of Well-Being: Theory, Perspectives and Practice. Hauppauge, NY: Nova Science Publishers (2013). p. 33–42. Available online at: https://login.proxy.library.rcsi.ie/login?qurl=https%3a%2f%2fsearch.ebscohost.com%2flogin.aspx%3fdirect%3dtrue%26db%3dpsyh%26AN%3d2013-28789-003%26site%3dehost-live (accessed February 21, 2022).

[88] Riley R Spiers J Chew-Graham CA Taylor AK Thornton GA Buszewicz M. “Treading water but drowning slowly”: what are GPs' experiences of living and working with mental illness and distress in England? A qualitative study. BMJ Open. (2018) 8:e018620. 10.1136/bmjopen-2017-018620 29724736 PMC5942433

[89] Spiers J Buszewicz M Chew-Graham CA Gerada C Kessler D Leggett N . Barriers, facilitators, and survival strategies for GPs seeking treatment for distress: a qualitative study. Br J Gen Pract J R Coll Gen Pract. (2017) 67:e700–8. 10.3399/bjgp17X692573 28893766 PMC5604834

[90] Spiers J Buszewicz M Chew-Graham CA Riley R. The experiences of general practitioner partners living with distress: an interpretative phenomenological analysis. J Health Psychol. (2018) 2018:1359105318758860. 10.1177/1359105318758860 29468904 PMC7479991

[91] Wainwright E Looseley A Mouton R O'Connor M Taylor G Cook TM. Stress, burnout, depression and work satisfaction among UK anaesthetic trainees: a qualitative analysis of in-depth participant interviews in the satisfaction and wellbeing in anaesthetic training study. Anaesthesia. (2019) 74:1240–51. 10.1111/anae.14694 31090927

[92] Daruvala R Ghosh M Fratazzi F Norzan SA Laha A Ahmed R . Emotional exhaustion in cancer clinicians: a mixed methods exploration. Indian J Med Paediatr Oncol. (2019) 40:111–20. 10.4103/ijmpo.ijmpo_168_17

[93] Nomura O Mishina H Kobayashi Y Ishiguro A Sakai H Kato H . Limitation of duty hour regulations for pediatric resident wellness: a mixed methods study in Japan. Medicine. (2016) 95:e4867. 10.1097/MD.0000000000004867 27631253 PMC5402596

[94] Loiselle ME. Academic physician burnout and transcendental meditation: a mixed methods randomized controlled trial. Dissert Abstracts Int Sect B Sci Eng. (2018) 79. 36702122 10.1097/CEH.0000000000000472

[95] Moola S Munn Z Tufanaru C Aromataris E Sears K Sfetcu R Currie M Qureshi R Mattis P Lisy K MP-F. Chapter 7: systematic reviews of etiology and risk - Joanna Briggs Institute Reviewers' manual. In: Joanna Briggs Institute Reviewer's Manual. JBI (2017).

[96] Robertson J Walkom EJ McGettigan P. Response rates and representativeness: a lottery incentive improves physician survey return rates. Pharmacoepidemiol Drug Saf . (2005) 14:571–7. 10.1002/pds.1126 15937989

[97] Chaukos D Chad-Friedman E Mehta DH Byerly L Celik A McCoy TH Jr . SMART-R: a prospective cohort study of a resilience curriculum for residents by residents. Acad Psychiatry J Am Assoc Dir Psychiatr Resid Train Assoc Acad Psychiatry. (2018) 42:78–83. 10.1007/s40596-017-0808-z 29098597

[98] Kroenke K Spitzer RL Williams JBW Löwe B. The patient health questionnaire somatic, anxiety, and depressive symptom scales: a systematic review. Gen Hosp Psychiatry. (2010) 32:345–59. 10.1016/j.genhosppsych.2010.03.006 20633738

[99] Zhang L Wang F Cheng Y Zhang P Liang Y. Work characteristics and psychological symptoms among GPs and community nurses: a preliminary investigation in China. Int J Qual Heal Care. (2016) 28:709–14. 10.1093/intqhc/mzw098 27614014

[100] Sun H Warner DO Macario A Zhou Y Culley DJ Keegan MT. Repeated cross-sectional surveys of burnout, distress, and depression among anesthesiology residents and first-year graduates. Anesthesiology. (2019) 131:668–77. 10.1097/ALN.0000000000002777 31166235

[101] Aish AM Wasserman D. Does Beck's Hopelessness Scale really measure several components? Psychol Med. (2001) 31:367–72. 10.1017/S0033291701003300 11232923

[102] Saunders JB Aasland OG Babor TF De La Fuente JR Grant M. Development of the alcohol use disorders identification test (AUDIT): WHO collaborative project on early detection of persons with harmful alcohol consumption-II. Addiction. (1993) 88:791–804. 10.1111/j.1360-0443.1993.tb02093.x 8329970

[103] Ewing JA. Detecting alcoholism: the CAGE questionnaire. JAMA. (1984) 252:1905–7. 10.1001/jama.1984.03350140051025 6471323

[104] Goerlich KS. The multifaceted nature of alexithymia - a neuroscientific perspective. Front Psychol. (2018) 9:1614. 10.3389/fpsyg.2018.01614 30210420 PMC6124373

[105] Vaismoradi M Turunen H Bondas T. Content analysis and thematic analysis: implications for conducting a qualitative descriptive study. Nurs Health Sci. (2013) 15:398–405. 10.1111/nhs.12048 23480423

[106] Biggerstaff D Thompson AR. Interpretative phenomenological analysis (IPA): a qualitative methodology of choice in healthcare research. Qual Res Psychol. (2008) 5:214–24. 10.1080/14780880802314304

[107] Mayring P. Qualitative content analysis. Companion Qual Res. (2004) 1:159–76.

[108] Koutsimani P Montgomery A Georganta K. The relationship between burnout, depression, and anxiety: a systematic review and meta-analysis. Front Psychol. (2019) 10:284. 10.3389/fpsyg.2019.00284 30918490 PMC6424886

[109] Dyrbye LN Thomas MR Shanafelt TD. Systematic review of depression, anxiety, and other indicators of psychological distress among US and Canadian medical students. Acad Med J Assoc Am Med Coll. (2006) 81:354–73. 10.1097/00001888-200604000-00009 16565188

[110] Lindeman S Läärä E Hakko H Lönnqvist J. A systematic review on gender-specific suicide mortality in medical doctors. Br J Psychiatry. (1996) 168:274–9. 10.1192/bjp.168.3.274 8833679

[111] Ten Have M De Graaf R Van Dorsselaer S Verdurmen J Van't Land H Vollebergh W . Incidence and course of suicidal ideation and suicide attempts in the general population. Can J Psychiatry. (2009) 54:824–33. 10.1177/070674370905401205 20047721

[112] Freedenthal S. Challenges in assessing intent to die: can suicide attempters be trusted? OMEGA. (2007) 55:57–70. 10.2190/5867-6510-3388-3517 17877081

[113] Louzon SA Bossarte R McCarthy JF Katz IR. Does suicidal ideation as measured by the PHQ-9 predict suicide among VA patients? Psychiatr Serv. (2016) 67:517–22. 10.1176/appi.ps.201500149 26766757

[114] Maslach C Jackson SE Leiter M. The Maslach Burnout Inventory Manual. (2015). Available online at: https://www.researchgate.net/publication/277816643 (accessed September 18, 2020).

[115] Mavroforou A Giannoukas A Michalodimitrakis E Mavroforou A Giannoukas A Michalodimitrakis E. Alcohol and drug abuse among doctors. Med Law. (2006) 25:611–25. 17263030

[116] Merlo LJ Singhakant S Cummings SM Cottler LB. Reasons for misuse of prescription medication among physicians undergoing monitoring by a physician health program. J Addict Med. (2013) 7:349–53. 10.1097/ADM.0b013e31829da074 24089039 PMC3790148

[117] Sibeoni J Bellon-Champel L Mousty A Manolios E Verneuil L Revah-Levy A. Physicians' perspectives about burnout: a systematic review and metasynthesis. J Gen Intern Med. (2019) 34:1578–90. 10.1007/s11606-019-05062-y 31147982 PMC6667539

[118] Lebares CC Hershberger AO Guvva E V Desai A Mitchell J Shen W . Feasibility of formal mindfulness-based stress-resilience training among surgery interns: a randomized clinical trial. JAMA Surg. (2018) 153:e182734. 10.1001/jamasurg.2018.2734 30167655 PMC6233792

[119] Panagioti M Panagopoulou E Bower P Lewith G Kontopantelis E Chew-Graham C . Controlled interventions to reduce burnout in physicians a systematic review and meta-analysis. JAMA Intern Med. (2017) 177:195–205. 10.1001/jamainternmed.2016.7674 27918798

[120] LaMontagne AD Keegel T Louie AM Ostry A Landsbergis PA. A systematic review of the job-stress intervention evaluation literature, 1990-2005. Int J Occup Environ Health. (2007) 13:268–80. 10.1179/oeh.2007.13.3.268 17915541

[121] Williams ES Rathert C Buttigieg SC. The personal and professional consequences of physician burnout: a systematic review of the literature. Med Care Res Rev. (2019) 2019:1077558719856787. 10.1177/1077558719856787 31216940

[122] Panagioti M Geraghty K Johnson J Zhou A Panagopoulou E Chew-Graham C . Association between physician burnout and patient safety, professionalism, and patient satisfaction a systematic review and meta-analysis. JAMA Intern Med. (2018) 178:1317–30. 10.1001/jamainternmed.2018.3713 30193239 PMC6233757

[123] Wang X Cheng Z. Cross-sectional studies: strengths, weaknesses, and recommendations. Chest. (2020) 158:S65–71. 10.1016/j.chest.2020.03.012 32658654

[124] Wiederhold BK Cipresso P Pizzioli D Wiederhold M Riva G. Intervention for physician burnout: a systematic review. Open Med. (2018) 13:253–63. 10.1515/med-2018-0039 29992189 PMC6034099

