# Supplementary material for: The relationship between physician burnout and depression, anxiety, suicidality and substance abuse: A mixed methods systematic review
Source: Front Public Health. 2023 Mar 30;11:1133484. doi: 10.3389/fpubh.2023.1133484 (PMC10098100; doi:10.3389/fpubh.2023.1133484)

## Supplemental File 1.

1a Search: Medline, Cinahl Plus and PsychInfo (via EBSCOHost)

| S1 | (MH “Occupational Stress) OR “occupational stress” |
| --- | --- |
| S2 | (MH “Burnout, Psychological”) OR (“Burnout, Professional”) OR “burnout” |
| S3 | S1 OR S2 |
| S4 | (MH “Depression’) OR “depression” OR (MH “depressive disorder” OR (MH “Depressive Disorder, Major”) |
| S5 | (MH “Anxiety” OR “anxiety” OR (MH “Anxiety, Disorders”) |
| S6 | (MH “Suicide”) OR “suicide” OR (MH “suicide, attempted”) OR (MH “Suicidal Ideation”) OR “suicidal” |
| S7 | (MH “Substance-Related Disorders”) OR “substance abuse” OR (MH “Drug Misuse”) OR ‘Drug addiction” |
| S8 | S4 OR S5 OR S6 OR S7 |
| S9 | “physician” OR (MH “Physicians”) OR “physicians” |
| S10 | “doctors” |
| S11 | (MH “Internship and Residency”) |
| S12 | S9 OR S10 OR S11 |
| S13 | S3 AND S8 AND S12 |

1b Search: Web of Science


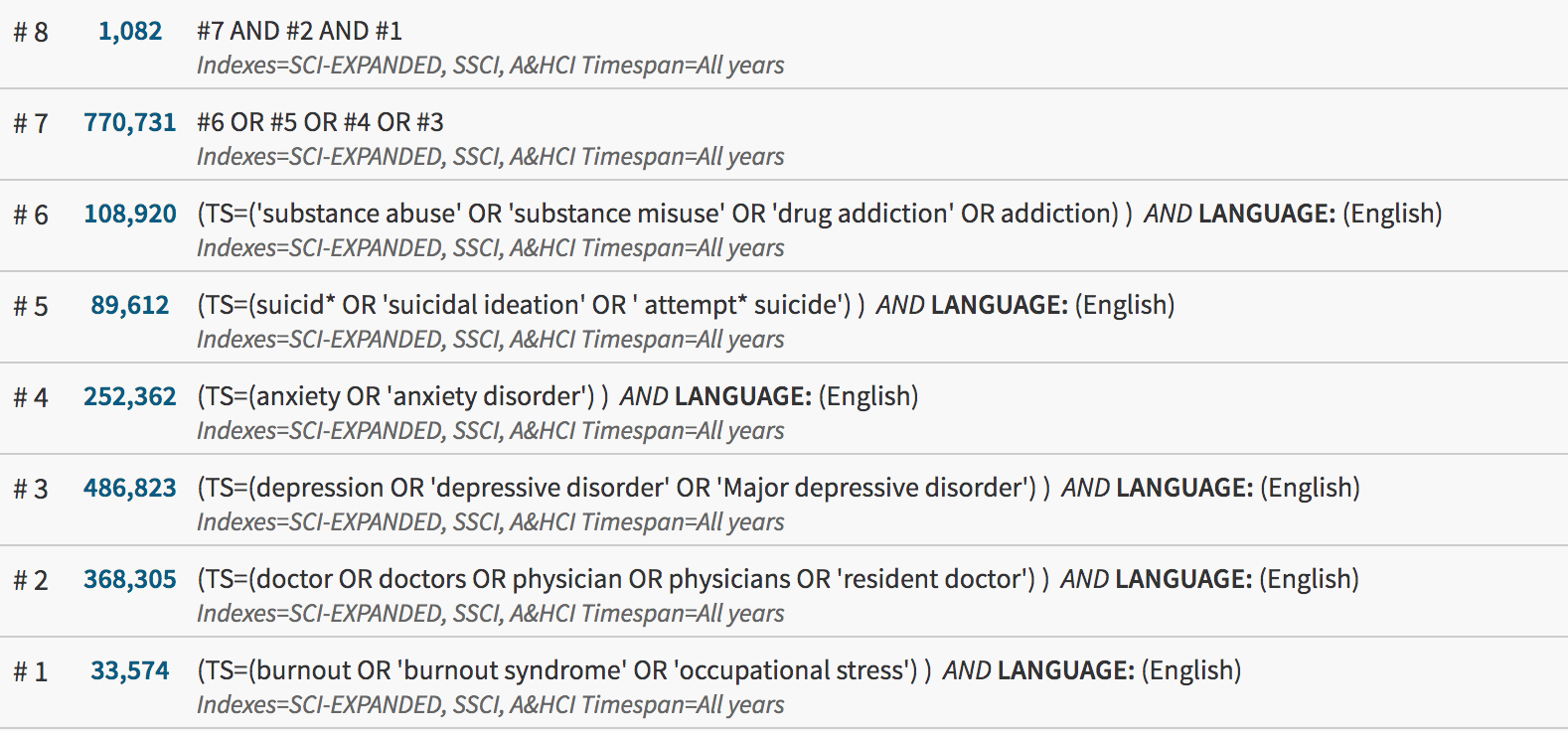

Supplement: Supplementary file 1 [file Table_1.docx]
