# Supplementary material for: The relationship between physician burnout and depression, anxiety, suicidality and substance abuse: A mixed methods systematic review
Source: Front Public Health. 2023 Mar 30;11:1133484. doi: 10.3389/fpubh.2023.1133484 (PMC10098100; doi:10.3389/fpubh.2023.1133484)
Supplement: Supplementary file 2 [file Table_2.DOCX]

**Supplemental Table** **1.**  **Quality Assessment of Quantitative Studies**

| Study | **Inclusion criteria clearly defined** | **Study subjects and setting defined** | **Exposure measure - valid and reliable** | **Objective standard criteria for measurement** | **Confounding factors identified** | **Strategies to deal with confounding identified** | **Outcomes measured in valid/reliable way** | **Appropriate statistical analysis** | **Overall assessment** |
| --- | --- | --- | --- | --- | --- | --- | --- | --- | --- |
| Carter 2019 | Y | N | Y | Y | Y | Y | Y | Y | **Moderate** |
| Chaukos 2017 | Y | Y | Y | Y | N | N | Y | Y | **Moderate** |
| Van der Heijden 2008 | Y | Y | Y | Y | N | N | Y | Y | **Moderate** |
| Faivre 2018 | Y | Y | Y | Y | Y | Y | Y | Y | **High** |
| Ferrari 2015 | Y | Y | Y | Y | Y | Y | Y | Y | **High** |
| Govardhan 2012 | Y | Y | Y | Y | N | N | Y | Y | **Moderate** |
| Juntunen 1988 | Y | Y | N | Y | Y | N | Y | U | **Low** |
| Karaoglu 2014 | Y | Y | Y | Y | Y | Y | Y | Y | **High** |
| Lazarescu 2018 | Y | Y | Y | Y | Y | N | Y | Y | **Moderate** |
| Lebares 2018 | Y | N | Y | Y | Y | Y | Y | Y | **Moderate** |
| Lebensohn 2013 | Y | Y | Y | Y | Y | Y | Y | Y | **High** |
| Lu 2015 | Y | Y | Y | Y | N | N | Y | Y | **Moderate** |
| Mohammed 2014 | N | Y | Y | Y | Y | N | Y | Y | **Moderate** |
| Nishimura 2019 | Y | Y | Y | Y | Y | Y | Y | Y | **High** |
| Nomura 2016 | Y | Y | Y | Y | Y | N | Y | N | **Moderate** |
| Ofei-Dodoo 2109 | Y | Y | Y | Y | Y | N | Y | N | **Moderate** |
| Pasqualucci 2019 | Y | Y | Y | Y | Y | Y | Y | Y | **High** |
| Siu 2012 | Y | Y | Y | Y | Y | Y | Y | Y | **High** |
| Sun 2012 | Y | N | Y | Y | Y | Y | Y | Y | **Moderate** |
| Talih 2016 | N | Y | Y | Y | Y | Y | Y | Y | **Moderate** |
| Willifred 2018 | Y | Y | Y | Y | Y | N | Y | Y | **Moderate** |
| Ashraf 2019 | Y | Y | Y | Y | N | N | Y | Y | **Moderate** |
| Boo 2018 | Y | Y | Y | Y | N | Y | Y | Y | **Moderate** |
| Bourne 2019 | Y | Y | Y | Y | N | Y | N | Y | **Moderate** |
| Daruvala 2019 | Y | Y | Y | Y | Y | N | Y | Y | **Moderate** |
| Haik 2017 | Y | Y | Y | Y | Y | N | Y | Y | **Moderate** |
| Hyman 2017 | Y | N | Y | Y | Y | Y | Y | Y | **Moderate** |
| Korkeila 2003 | Y | Y | Y | Y | N | N | N | Y | **Low** |
| Janko 2019 | Y | Y | Y | Y | N | Y | Y | Y | **Moderate** |
| Khan 2018 | Y | Y | Y | Y | Y | Y | Y | Y | **High** |
| Mampuya 2017 | N | Y | Y | Y | N | Y | Y | Y | **Moderate** |
| Shanfelt 2011 | Y | Y | Y | Y | Y | Y | Y | Y | **High** |
| Thomassen 2001 | Y | Y | Y | Y | N | N | Y | Y | **Moderate** |
| Wurm 2016 | Y | Y | Y | Y | N | N | Y | Y | **Moderate** |
| Yilmaz 2018 | N | Y | Y | Y | N | N | Y | U | **Low** |
| Zhang 2019 | Y | Y | Y | Y | Y | Y | Y | Y | **High** |
| Zhou 2016 | Y | Y | Y | Y | N | N | Y | Y | **Moderate** |
| Faivre 2019 | Y | Y | Y | Y | Y | Y | Y | Y | **High** |
| Looseley 2019 | Y | Y | Y | Y | Y | Y | Y | Y | **High** |
| Whitely 1989 | Y | Y | U | Y | Y | Y | Y | Y | **Moderate** |
| Becker 2006 | Y | Y | Y | Y | Y | Y | Y | Y | **High** |
| Bernburg 2016 | Y | Y | U | Y | Y | Y | Y | Y | **Moderate** |
| Iorga 2017 | Y | Y | Y | Y | N | Y | N | Y | **Moderate** |
| Pompili 2010 | Y | Y | Y | Y | N | Y | Y | Y | **Moderate** |
| Sahin 2019 | Y | U | Y | Y | N | N | Y | Y | **Low** |
| Toral-Villanueva 2009 | Y | Y | Y | Y | Y | Y | Y | Y | **High** |
| Oreskovich 2012 | Y | Y | Y | Y | Y | Y | Y | Y | **High** |
| Williamson 2018 | Y | Y | Y | Y | N | N | Y | Y | **Moderate** |
| Oreskovich 2015 | Y | Y | U | Y | Y | Y | Y | Y | **Moderate** |
| Mikalauskas 2018 | Y | U | Y | Y | N | N | Y | Y | **Low** |
| Pederson 2016 | Y | Y | Y | Y | U | Y | Y | Y | **Moderate** |
| Rath 2015 | Y | Y | Y | Y | Y | Y | Y | Y | **High** |
| Tateno 2018 | Y | Y | Y | Y | N | N | Y | Y | **Moderate** |

Y=Yes, N=No, U=Unclear
