# Supplementary material for: The relationship between physician burnout and depression, anxiety, suicidality and substance abuse: A mixed methods systematic review
Source: Front Public Health. 2023 Mar 30;11:1133484. doi: 10.3389/fpubh.2023.1133484 (PMC10098100; doi:10.3389/fpubh.2023.1133484)
Supplement: Supplementary file 3 [file Table_3.DOCX]

Supplemental Table 2 Odds Ratios of Burnout as a Risk Factor for Depression

| Study ID | Number of participants | Association | Odds ratio |
| --- | --- | --- | --- |
| Faivre 2018 | 107 | Depressive symptoms as a risk factor for burnout | OR 19.3 (p =0.0048) |
| Lazerescu 2018 | 242 | Moderate/severe burnout as a risk factor for high depression score | OR 2.96 (p < 0.0001) |
| Pasqualucci 2019 | 606 | Burnout as a risk factor for depression | OR 2.7 (p = 0.001) |
| Boo 2018 | 303 | High burnout as a risk factor for depression | OR 0.89 (p = 0.007) |
| Bourne 2018 | 3102 | Burnout as a risk factor for self-reported depression | OR 4.05 (95% CI 3.26 – 5.04) |
| Korkeila 2003 | 298 | Burnout score as risk factor for self-reported depression | OR 5 (95% CI 2.2-11) |
| Janko 2019 | 177 | Highest quartile of burnout as a risk factor for moderate/severe depression | OR 2 (p < 0.01) |
| Wurm 2016 | 5897 | Risk of suffering from severe depression with mild/moderate or severe burnout symptoms | Mild OR 2.99 ( 95% CI 2.21 – 4.06)  Moderate OR 10.14 (95% CI 7.6 – 13.6)  Severe OR 46.8 (95% CI 35.3 – 63.2) |
| Zhang 2019 | 159 | Job burnout as a risk factor for depressive symptoms | OR 10.68 (p > 0.0001) |
| Faivre 2019 | 441 | Depressive symptoms as a risk factor for job burnout | OR 6.3 (p = 0.0006) |
| Toral-Villanueva 2009 | 312 | Current depression as a risk factor for burnout | OR 5.6 (95% CI 3.3–9.5) |
| Mikalauskas 2018 | 220 | Positive depression screen as a risk factor for burnout | OR 10.3 (p < 0.01) |
| Rath 2015 | 369 | Positive screen for depression as a risk factor for burnout | OR 7.37 (p < 0.000001) |
