# Supplementary material for: The relationship between physician burnout and depression, anxiety, suicidality and substance abuse: A mixed methods systematic review
Source: Front Public Health. 2023 Mar 30;11:1133484. doi: 10.3389/fpubh.2023.1133484 (PMC10098100; doi:10.3389/fpubh.2023.1133484)
Supplement: Supplementary file 4 [file Table_4.DOCX]

Supplemental Table 3 Correlation between Overall Burnout Score and Depression

| Study ID | Number of participants | Variable measured | Correlation |
| --- | --- | --- | --- |
| Talih 2016 | 118 | Severity of depression | R = 0.72 (p < 0.001) |
| Korkeila 2003 | 298 | Overall Depression score | R=0.41, p<0.001 |
| Wurm 2016 | 5897 | Overall Depression Score | R = 0.74, p< 0.001 |
