# Supplementary material for: The relationship between physician burnout and depression, anxiety, suicidality and substance abuse: A mixed methods systematic review
Source: Front Public Health. 2023 Mar 30;11:1133484. doi: 10.3389/fpubh.2023.1133484 (PMC10098100; doi:10.3389/fpubh.2023.1133484)
Supplement: Supplementary file 5 [file Table_5.DOCX]

Supplemental Table 4 Association Between Depression and Burnout Domains as Odds Ratios

**Emotional Exhaustion**

| Study ID | Number of Participants | Association | Odds Ratio |
| --- | --- | --- | --- |
| Lazarescu 2018 | 242 | Emotional exhaustion score as a risk factor for high depression score | OR 4.7 (p < 0.001) |
| Lebares 2018 | 566 | Emotional exhaustion as a risk factor for moderate/severe depression | OR 4.81 (p < 0.001) |
| Daruvala 2019 | 114 | High depression score as a risk factor for high emotional exhaustion score | OR 2.7 p = 0.09 |
| Korkeila 2003 | 289 | Self-reported depression as a risk factor for high EE score | OR 6.9 (95 CI 1.4 - 34.1) |

**Depersonalisation**

| Study ID | Number of Participants | Association | Odds ratio |
| --- | --- | --- | --- |
| Lazarescu 2018 | 242 | Lack of Personal Accomplishment as a risk factor for depression | OR 2.2 (p = 0.003) |

**Personal Accomplishment**

| Study ID | Number of Participants | Association | Odds Ratio |
| --- | --- | --- | --- |
| Lebares 2018 | 566 | Depersonalisation as a risk factor for moderate/severe depression | OR 2.36 (p < 0.0009) |
