# Supplementary material for: The relationship between physician burnout and depression, anxiety, suicidality and substance abuse: A mixed methods systematic review
Source: Front Public Health. 2023 Mar 30;11:1133484. doi: 10.3389/fpubh.2023.1133484 (PMC10098100; doi:10.3389/fpubh.2023.1133484)
Supplement: Supplementary file 6 [file Table_6.DOCX]

Supplemental Table 5 Correlation between Burnout Scores and Depression Scores

**Emotional Exhaustion**

| Study ID | Number of participants | Variables measured | Correlation |
| --- | --- | --- | --- |
| Sahin 2019 | 158 | Overall Depression Scores | R = 0.516 (p < 0.00001) |
| Lebensohn 2013 | 168 | Overall depression score | R = 0.584 (p <0.001) |
| Mohammed 2014 | 84 | Severity of depression | R=0.61 (p< 0.001) |
| Nishimura 2019 | 39 (T1)  27 (T2)  21 (T3) | Overall Depression Score | r = .615, p < .01 (T1)  r = .706, p < .01 (T2)  r = .601, p < .01 (T3) |
| Khan 2018 | 593 | Depressive symptoms | R = 0.61 (p < 0.01) |
| Mampuya 2017 | 87 | Psychological morbidity (depression) | R= 0.16 (p <0.01) |
| Yilmaz | 343 | Average depression points | R=0.41 (p <0.0001) |
| Karaoglu 2014 | 74 | Overall depression score | R = 0.65 |

**Depersonalisation**

| Sahin 2019 | 158 | Overall depression scores | R = 0.311 (p < 0.00001) |
| --- | --- | --- | --- |
| Lebensohn 2013 | 168 | Overall depression score | R =0.518 (p < 0.001) |
| Mohammed 2014 | 84 | Severity of depression | R= 0.63 (p < 0.001) |
| Nishimura 2019* | 39 (T1)  27 (T2)  21 (T3) | Overall Depression Score | r = .279, p < .09 (T1)  r = .047, p < .82 (T2)  r = .176, p < .45 (T3) |
| Khan 2018 | 593 | Depressive Symptoms | R = 0.40 (p , 0.01) |
| Mampaya 2017* | 87 | Psychological morbidity (depression) | R = -0.05 (p = 0.48) |
| Yilmaz* | 343 | Average depression points | R= 0.33 |

**Personal Accomplishment**

| Sahin 2019 | 158 | Overall depression scores | R = -0.218 (p< 0.00001) |
| --- | --- | --- | --- |
| Mohammed 2014 | 84 | Severity of depression | R = -0.56 (p < 0.001) |
| Mampuya 2017 | 87 | Psychological morbidity (depression) | R = -0.1 (p < 0.01) |
| Yilmaz* | 343 | Average depression points | R = -0.09 |

**(*) not statistically significant**
