# Supplementary material for: The relationship between physician burnout and depression, anxiety, suicidality and substance abuse: A mixed methods systematic review
Source: Front Public Health. 2023 Mar 30;11:1133484. doi: 10.3389/fpubh.2023.1133484 (PMC10098100; doi:10.3389/fpubh.2023.1133484)
Supplement: Supplementary file 7 [file Table_7.DOCX]

Supplemental Table 6 Quality Assessment of Qualitative Studies

| **Study ID** | **Statement of Aims** | **Qualitative Methodology Appropriate** | **Research Design** | **Recruitment Strategy** | **Data Collection** | **Relationship between researcher &participants** | **Ethical Issues** | **Rigorous Data Analysis** | **Clear Statement of Findings** | **How Valuable is Research** | **Overall Score** |
| --- | --- | --- | --- | --- | --- | --- | --- | --- | --- | --- | --- |
| Hamader 2013 | Y | Y | U | U | U | N | N | N | N | Y | **Low** |
| Daruvala 2019 | Y | Y | N | Y | Y | Y | Y | Y | Y | Y | **High** |
| Loiselle 2018 | Y | Y | Y | U | Y | Y | U | Y | Y | Y | **Moderate** |
| Riley 2018 | Y | Y | Y | Y | Y | Y | U | Y | Y | Y | **High** |
| Spiers 2017 | Y | Y | Y | N | Y | U | Y | Y | Y | Y | **Moderate** |
| Spiers 2018 | Y | Y | Y | U | Y | U | Y | Y | Y | Y | **Moderate** |
| Wainwright 2019 | Y | Y | Y | Y | Y | Y | Y | Y | Y | Y | **High** |

Y=Yes, N=No, U=Unclear
